# Community-based interventions to promote paternal mental wellbeing and prevent poor mental health during the perinatal period: a mixed methods systematic review

**DOI:** 10.3389/fpubh.2026.1823143

**Published:** 2026-08-14

**Authors:** Sharin Baldwin, Vicky Gilroy, Sally Kendall

**Affiliations:** 1 Centre for Health Services Studies, University of Kent, Canterbury, United Kingdom; 2 Innovation and Research, Institute of Health Visiting, London, United Kingdom; 3 School of Nursing and Midwifery, Western Sydney University, Penrith, NSW, Australia

**Keywords:** community, father, health visitor, mental health, mental wellbeing, perinatal period, prevention, promotion

## Abstract

**Background:**

Paternal mental health during the perinatal period is increasingly recognized as a public health priority. Poor paternal mental health can negatively affect fathers, partners, and children, while mentally well and engaged fathers contribute positively to child development. However, evidence on effective community-based interventions remains limited.

**Objectives:**

The aim of this systematic review was to identify and synthesize the best available evidence on community-based interventions that promote good mental health or prevent poor mental health among fathers in the perinatal period. By integrating quantitative and qualitative data, it sought to inform public health practice and policy with a comprehensive understanding of intervention effectiveness and acceptability.

**Methods:**

The review included quantitative, qualitative, and mixed methods studies published in English from 2009 to 2025. The JBI approach to critical appraisal, study selection, data extraction and data synthesis was used. Quantitative data was “qualitized” and synthesized with qualitative data using a convergent integrated approach.

**Results:**

Nineteen papers representing 18 studies met the eligibility criteria. All studies evaluated community-accessible interventions including psychosocial, psychoeducational, peer support, and digital or technology-assisted approaches delivered to fathers individually, within couples, or in group settings. Twelve studies were quantitative, three mixed methods, and three were qualitative. Studies were conducted across 11 countries. In total, 39 qualitative findings and 11 qualitized findings were synthesized into six integrated findings: 1) Reduced social isolation and increased feelings of connection and belonging; 2) Validation, emotional reassurance, and access to safe, non-judgmental spaces; 3) Strengthened paternal identity, confidence, and readiness for fatherhood; 4) Enhanced father–infant bonding and caregiving involvement; 5) Improved partner relationships and co-parenting through better communication and confidence; 6) Increased knowledge, coping strategies, and mental health literacy.

**Conclusion:**

While community-based interventions show mixed effects on standardized mental health outcomes, integrated evidence indicates meaningful psychosocial and relational benefits for fathers. Promoting paternal mental health during the perinatal period may therefore depend less on short-term symptom reduction and more on strengthening social connection, identity, and confidence, highlighting the need for broader outcome frameworks and more rigorous evaluation.

## Introduction

Men's mental health during the perinatal period is increasingly recognized as a crucial public health issue due to its impact on fathers, partners, and child development. Poor paternal mental health is associated with adverse cognitive, social, and emotional outcomes in children and an increased the risk of later depression (1–4). A Welsh cohort study reported that paternal depression increased children's risk of depression and had a stronger negative effect on educational outcomes than maternal depression (5).

Conversely, positive paternal mental health is associated with improved child empathy, self-esteem, behavior, and academic outcomes (6–10), as well as enhanced partner support (11). However, focusing solely on the treatment of mental illness is insufficient. Mental wellbeing encompasses broader dimensions including emotional resilience, coping, role confidence, social connection, relational functioning, and adaptation to fatherhood (12). Fathers may therefore experience distress or reduced wellbeing without meeting diagnostic thresholds, highlighting the need for both prevention and promotion-focused approaches. This aligns with UK policy priorities emphasizing preventive, community-based mental healthcare and early intervention across the life course (13, 14).

Increasing emphasis within UK perinatal mental health and men's health policy on prevention, early intervention, father inclusion, and community-based support informed the focus and design of this review (13–15). In particular, the review was designed to examine interventions operating within community contexts and to explore not only treatment outcomes, but also the promotion of positive mental wellbeing and prevention of poor mental health among fathers during the perinatal period.

Health visitors in the UK, who are specialist community public health nurses, are uniquely positioned to support fathers' mental wellbeing during the perinatal period because they have universal contact with families from pregnancy until a child reaches 5 years of age. Despite this strategic position, there is currently limited evidence about which approaches are most effective in promoting positive mental wellbeing or preventing mental health problems among men during the perinatal period.

### Current knowledge and gaps

The transition to fatherhood involves major emotional, psychological, relational, and social changes that can increase vulnerability to poor mental health and reduced wellbeing (16, 17). Anxiety and depression are among the most commonly studied mental health difficulties and can affect fathers' wellbeing and their ability to support partners and children. Paternal anxiety affects up to 16% of fathers antenatally and rises to 18% postnatally (18). A meta-analysis of 23 studies found a prevalence of 10.7% during the perinatal period, which is higher than global male anxiety rates (19, 20).

Rates of paternal depression range from 4–25% among first-time fathers (21–23). A meta-analysis of 43 studies from 2010 reported a prevalence rate of depression in men pre- and postnatally as around 10.4% (22). Another later meta-analysis published in 2016, reported an estimated rate of paternal depression of 8.4% from pregnancy through the first postpartum year (23). However, the prevalence of depression can be much higher when the partner is also experiencing mental health challenges. Children with two depressed parents are at increased risk of poor development outcomes (24). Less common but significant conditions such as post-traumatic stress disorder (PTSD), obsessive-compulsive disorder (OCD), psychosis, and bipolar disorder can further impair family functioning.

Despite the recognition of fatherhood as a major life transition, evidence on effective interventions to support fathers' mental health and wellbeing during the perinatal period remains limited (16, 25). In a recent scoping review, Watkins et al. (26) aimed to explore fathers' concerns and challenges impacting their mental health and wellbeing during the transition to fatherhood, as well as their experiences with antenatal programs and psychological/social support. The findings primarily focused on fathers' mental health, with less attention on available interventions. The review identified limited data on fathers' experiences with perinatal support and emphasized the need for further research in this area to better inform future interventions. The review, which only included qualitative studies, concluded that there is a significant gap in understanding fathers' support needs and stressed the importance of developing inclusive, evidence-based interventions to improve fathers' mental health during the transition to fatherhood.

Chua and Shorey (27) conducted a systematic review to assess the effectiveness of mobile application-based perinatal interventions in improving parenting outcomes such as parenting self-efficacy, anxiety, depression, stress, social support, and parent-child bonding. The review focused on interventions delivered through mobile apps, excluding those based on text messaging, phone calls, video calls, or formal therapy sessions. Only randomized controlled trials (RCTs), cluster RCTs, and controlled clinical trials (CCTs) were included. The review found that mobile application-based perinatal interventions were effective in improving parents' wellbeing, especially during the COVID-19 pandemic. However, only three of 12 studies included fathers, limiting generalizability.

Another systematic review by Rominov et al. (28), of intervention programmes to prevent or treat paternal mental illness in the perinatal period included 11 studies: five of which described psychosocial programmes (emphasizing skills, knowledge, emotional wellbeing, and social wellbeing related to parenting), three focused on the effects of massage techniques (partner massage and infant massage), and three which used couple-based sessions (focused on the couple relationship and co-parenting). Six of the eight randomized controlled trials included did not provide adequate information on randomization processes and risk of bias could not be ruled out. The review found significant positive effects on fathers' mental health outcomes (such as stress, depression, anxiety, anger, and self-esteem) associated with psychosocial and massage-based interventions. However, couple-based interventions did not show significant improvements in paternal mental health. The authors noted limitations, including poor study design reporting, variability in outcome measures, and limited statistical analysis.

Other systematic reviews have investigated interventions for the treatment of paternal perinatal depression (29, 30) or those focusing on fatherhood (promoting positive parenting, co-parenting, and father-child relationships) (31), rather than the promotion of good mental health or prevention of poor mental health among men in the perinatal period. Overall, there remains a lack of evidence on community-based interventions that promote wellbeing, coping, and psychosocial functioning or prevent deterioration in paternal mental health during the perinatal period.

Searches of major databases identified no completed systematic reviews addressing this specific gap. Two ongoing reviews (32, 33) focus on RCTs of mobile or early parenting interventions but do not include mixed-methods evidence.

### Conceptual definitions

Paternal mental wellbeing is conceptualized as a multidimensional construct encompassing emotional wellbeing, coping capacity, social connectedness, relational functioning, role adaptation, and psychological adjustment during the transition to fatherhood alongside the absence of mental illness (12). Poor mental health refers to psychological distress and reduced psychosocial functioning during the perinatal period. Although outcomes such as parenting confidence, paternal identity, father–infant bonding, co-parenting, and relationship quality are conceptually distinct from mental illness symptoms such as depression and anxiety, they are closely interconnected and may influence fathers' overall wellbeing during the perinatal period. This review adopts a broader perspective that extends beyond the absence of mental illness to include positive psychosocial and relational functioning. Community-based interventions include those delivered outside inpatient or specialist psychiatric settings, such as primary care, home-visiting, peer support, voluntary sector services, group programmes, and digital interventions.

## Review question

The review question was: What community-based interventions promote mental wellbeing or prevent poor mental health among men in the perinatal period?

The aim of this systematic review was to strengthen the evidence base for community-based interventions that promote paternal mental wellbeing or prevent poor mental health during the perinatal period. A mixed-methods approach was used to integrate quantitative and qualitative evidence, enabling analysis of both intervention effectiveness and fathers' reported experiences. This approach was selected to capture the complexity of interventions and provide a more comprehensive understanding than either data type could offer in isolation.

Outcomes of interest included paternal mental health and wellbeing outcomes measured quantitatively or qualitatively. These included symptom-focused outcomes such as depression, anxiety, stress, and psychological distress, alongside psychosocial, relational, and wellbeing-related outcomes including coping, confidence, paternal identity, social connectedness, father–infant and partner relationships, mental health literacy, and help-seeking experiences.

The review design and inclusion criteria were aligned with UK policy priorities emphasizing prevention, early intervention, and community-based support for paternal mental health.

### Inclusion criteria

#### Population

This review considered studies that included fathers (defined as individuals who had parental responsibility for a child, including biological fathers, adoptive fathers, or stepfathers) during the perinatal period (from conception to 12 months postpartum).

Certain groups of fathers may have specific mental health needs during the perinatal period; therefore, the following were excluded:

- Studies involving fathers who experienced bereavement following neonatal death, stillbirth, pregnancy loss, or sudden infant death.
- Studies involving fathers whose infants were born pre-maturely (<37 weeks' gestation).
- Studies involving fathers of children with terminal or long-term conditions.
- Studies involving fathers with severe mental illness.

#### Phenomena of interest

This review considered studies that investigated community-based interventions that promoted good mental health, or prevented poor mental health, among men in the perinatal period. This included psychosocial, health promotion, peer support or technology-based interventions, delivered to individuals, couples or groups.

The extracted outcomes were organized into three broad domains:

1. Symptom-focused mental health outcomes, including depression, anxiety, stress, and psychological distress.
2. Psychosocial and relational wellbeing outcomes, including coping, confidence, paternal identity, social connectedness, emotional wellbeing, and partner or father–infant relationships.
3. Engagement, acceptability, and preventative processes, including intervention engagement, retention, perceived usefulness, help-seeking, mental health literacy, and accessibility.

#### Context

This review considered studies that investigated interventions that were accessible to fathers in the community settings (including technology-based interventions) and offered in the perinatal period (from the point of conception to 1 year after birth). This time period aligns with the period during which health visitors work with families, providing opportunities for early identification and preventive intervention to support paternal mental wellbeing.

#### Types of studies

This review considered quantitative, qualitative and mixed methods studies. Quantitative studies included both experimental and quasi-experimental study designs, including randomized controlled trials, non-randomized controlled trials, before and after studies and interrupted time-series studies. In addition, analytical observational studies including prospective and retrospective cohort studies, case-control studies and analytical cross-sectional studies were considered for inclusion. This review also considered descriptive observational study designs including case series, individual case reports and descriptive cross-sectional studies for inclusion.

Qualitative studies included, but not limited to, designs such as phenomenology, grounded theory, ethnography and action research. The review also considered qualitative data reported within quantitative surveys for inclusion, where open questions relating to the phenomena of interest were asked.

Mixed method studies were only considered if data from the quantitative or qualitative components could be clearly extracted.

## Methods

The proposed systematic review was conducted in accordance with the JBI methodology for Mixed Methods Systematic Reviews (65). The protocol was also registered with PROSPERO (ID:1020109).

### Search strategy

The search strategy aimed to identify both published and unpublished studies. An initial limited search of MEDLINE and CINAHL was undertaken to identify articles on the topic. The text words contained in the titles and abstracts of relevant articles, and the index terms used to describe the articles were used to develop a full search strategy (Appendix 1). The search strategy, including all identified keywords and index terms were adapted for each included information source. The reference list of all studies selected for critical appraisal were screened for additional studies.

Studies published in English were considered for inclusion as resources for translation were not available to the reviewers. Searches were conducted between January and August 2025, and studies published from 2009 onwards were considered for inclusion to reflect the shift in UK policy, when the national Healthy Child Programme placed greater emphasis on parenting support, specifically focusing on strengthening couple relationships, engaging fathers, and supporting the transition to parenthood for both mothers and fathers (34, 35).

### Information sources

The databases searched included MEDLINE (EBSCO), CINAHL complete (EBSCO), APA PsycINFO (EBSCO), British Nursing Index (EBSCO), Scopus (Ovid), Embase (Ovid), and Web of Science.

A search of The Fatherhood Institute website was conducted alongside a manual search on Google Scholar.

The search for unpublished studies such as theses and dissertations included ProQuest Dissertations and Theses Global and World Cat dissertations and Theses (OCLC).

#### Study selection

The initial database searches and citation tracking was performed by the first author (SB). Following the search, all identified citations were collated and uploaded into Covidence (Veritas Health Innovation, Melbourne, Australia), an online systematic review management platform. Covidence was used to manage the screening and review process, including title and abstract screening, full-text review, and recording decisions. Duplicates were automatically removed. Titles and abstracts were then screened by the first author (SB) for assessment against the inclusion criteria for the review. Ten percent of titles and abstracts were independently screened by the second author (VG) to ensure consistency and reliability in study selection. This proportion was considered appropriate given resource and time constraints and is consistent with approaches used in systematic reviews where full dual screening is not feasible.

Titles and abstracts were then screened by two reviewers for assessment against the inclusion criteria for the review (SB, VG). Potentially relevant studies were retrieved in full and assessed in detail against the inclusion criteria by the two reviewers independently (SB, VG). Full text studies that did not meet the inclusion criteria were excluded and any disagreements that arose between the reviewers were resolved through discussion.

#### Assessment of methodological quality

Eligible studies were critically appraised by the same two independent reviewers (SB, VG) for methodological quality using standardized critical appraisal instruments from JBI SUMARI (36).

It was not necessary to contact the authors of the included papers. Any discrepancies between reviewers were resolved through detailed discussion until consensus was reached.

All included studies underwent critical appraisal using the Joanna Briggs Institute (JBI) Critical Appraisal Checklist appropriate to each study design (37). In line with the pre-defined protocol, a provisional threshold of 50% of applicable criteria was established *a priori* to guide potential exclusion of methodologically weak studies. This threshold was intended as a pragmatic and transparent decision-making aid rather than a formal quality scoring system, recognizing that JBI tools are not designed to generate numerical scores or summary ratings (37). Instead, appraisal decisions were informed primarily by domain-level judgements relating to methodological quality and risk of bias. Although percentage-based interpretations of JBI tools may oversimplify complex methodological considerations, the structured approach supported consistency and transparency in appraisal and reporting (38). All included studies met or exceeded the pre-defined threshold; therefore, no studies were excluded on this basis.

#### Data extraction

Quantitative and qualitative data were extracted from included studies by two independent reviewers (SB, VG) using the standardized JBI data extraction tools in JBI SUMARI. The data extracted included specific details about the population, study methods, the phenomenon of interest, context and outcomes of relevance to the review question(s). Specifically, quantitative data comprised data-based outcomes of descriptive and/or inferential statistical tests. In addition, qualitative data comprised of themes or subthemes with corresponding illustrations, which were assigned a level of credibility by the first reviewer (SB) using the three levels as described by the standardized JBI qualitative data extraction tool:

i) Unequivocal (U)-evidence beyond reasonable doubt which may include conclusions that are matter of fact, directly reported/observed and not open to challenge.
ii) Credible (C)-those conclusions that are, albeit interpretations, plausible in light of the data and theoretical framework.
iii) Not Supported-when the findings are not supported by the data.

These were then discussed amongst the three reviewers (SB, VG, SK) resulting in general consensus with allocation of these levels (Appendix 2). ‘Not Supported' data was excluded from the synthesis of data.

#### Data transformation

The quantitative data was converted into ‘qualitized data'. This involved transformation into textual descriptions or narrative interpretation of the quantitative results from experimental and observational studies (including the quantitative component of mixed methods studies), in a way that answered the review questions by repeated detailed examination (Appendix 3).

#### Data synthesis and integration

The convergent integrated approach according to the JBI methodology for mixed methods systematic review using JBI SUMARI was used in this review (36). This involved assembling the ‘qualitized' data with the qualitative data. Assembled data were categorized and pooled together based on similarity in meaning to produce a set of integrated findings in the form of line of action statements.

### Service User Involvement

Three fathers were consulted to support interpretation of the review findings, particularly in relation to the relevance and meaning of psychosocial and relational outcomes for men. Their input also contributed to shaping the recommendations, ensuring that suggested approaches reflected lived experience perspectives alongside the research evidence. The fathers will further support dissemination of the findings through the development of accessible summary materials.

## Results

### Study inclusion

**Figure 1:**
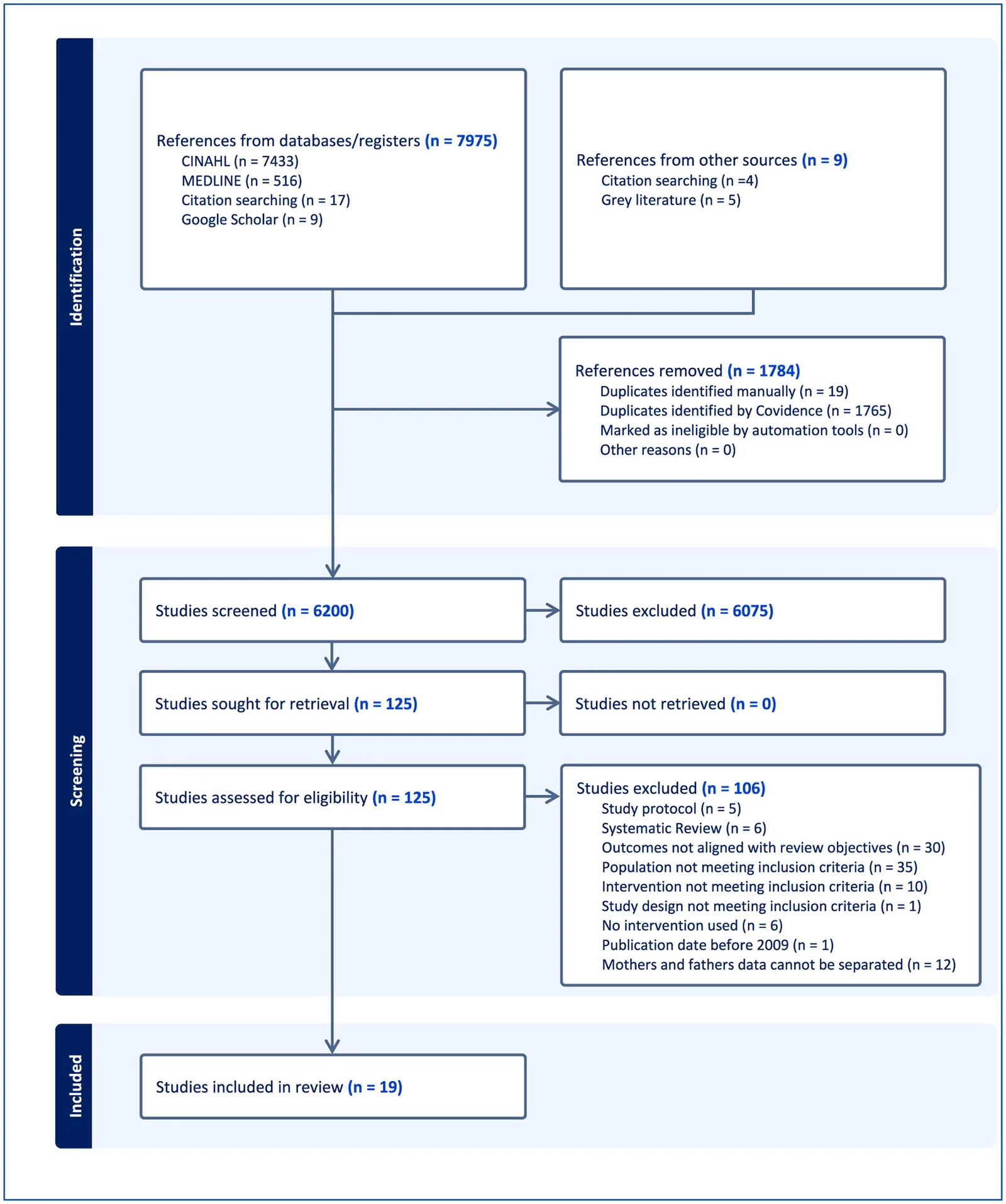
PRISMA flow diagram of search and study selection process.

The literature search initially identified 7,975 records through database searching and nine through citation searching and gray literature, resulting in 7,984 potentially relevant records. On further examination of the study titles, 1,784 of these records were excluded for duplication. The titles and abstracts were reviewed for the remaining 6,200 records, and a further 6,075 were excluded for various reasons, including, opinion papers, those not focusing on the phenomena of interest or not meeting the inclusion criteria. The remaining 125 articles were retrieved for a full review, following which, 106 were excluded based on the agreed inclusion and exclusion criteria (research protocol = 5, systematic review = 6, outcomes not aligned with review objectives = 30, populations outside the inclusion criteria = 35, interventions not meeting review definition of community-based support = 10, study design not meeting inclusion criteria = 1, intervention not used = 6, publication date before 2009 = 1, mothers and fathers data separated = 12), leaving 19 articles to be examined for methodological quality (see Figure 1).

### Methodological quality

Although 19 papers were identified, these represented 18 unique studies, as two papers reported outcomes from the same study (39, 40), which used a single-group pre–post pilot design with reliable change analysis and an embedded qualitative component reported separately. Methodological quality of the 19 included papers was determined by the relevant JBI critical appraisal instrument. The JBI Critical Appraisal Checklist for Qualitative Research provided a framework for scoring the quality of qualitative studies and qualitative components of mixed methods studies, whereas the JBI Critical Appraisal Checklist for Randomized Controlled Trials, and Quasi-Experimental Studies were used for quantitative studies and quantitative components of mixed methods studies (see Tables 1–3).

**Table 1:** Critical appraisal results for included studies using the JBI critical appraisal checklist for randomized controlled trials and RCT component of mixed methods studies.

References	Q1	Q2	Q3	Q4	Q5	Q6	Q7	Q8	Q9	Q10	Q11	Q12	Q13	Total
1. Ammerman et al. (41)	Y	N	Y	N	N	N	Y	Y	Y	Y	Y	Y	Y	9/13
6. Kaner et al., (47)^*^	Y	Y	Y	N	Y	Y	Y	Y	Y	Y	Y	Y	Y	12/13
7. Kavanagh et al. (48)	Y	Y	Y	N	Y	Y	Y	Y	Y	Y	Y	Y	Y	12/13
9. Li et al. (42)	Y	N	Y	N	N	N	Y	Y	N	Y	Y	Y	Y	8/13
10. Mihelic et al. (50)	Y	Y	Y	N	N	N	Y	Y	Y	Y	Y	Y	Y	10/13
11. Missler et al. (51)	Y	Y	Y	N	N	N	Y	Y	Y	Y	Y	Y	Y	10/13
12. Mohammadpour et al. (43)	Y	Y	Y	N	N	N	Y	Y	Y	Y	Y	Y	Y	10/13
14. Shorey et al. (44)	Y	Y	Y	N	U	U	Y	U	U	Y	Y	Y	Y	8/13
15. Shorey et al. (52)	Y	Y	Y	U	N	U	Y	Y	Y	Y	Y	Y	Y	10/13

Y, yes; N, no; NA, not applicable; U, unclear.

^*^Mixed methods studies, where two different appraisal tools have been used.

(1) Was true randomization used for assignment of participants to treatment groups? (2) Was allocation to treatment groups concealed? (3) Were treatment groups similar at the baseline? (4) Were participants blind to treatment assignment? (5) Were those delivering treatment blind to treatment assignment? (6) Were outcomes assessors blind to treatment assignment? (7) Were treatment groups treated identically other than the intervention of interest? (8) Was follow up complete, if not, were differences between groups adequately described and analyzed? (9) Were participants analyzed in the groups to which they were randomized? (10) Were outcomes measured in the same way for treatment groups? (11) Were outcomes measured in a reliable way? (12) Was appropriate statistical analysis used? (13) Was the trial design appropriate, and any deviations from the standard RCT design (individual randomization, parallel groups) accounted for in the conduct and analysis of the trial?.

**Table 2:** Critical appraisal results for included studies using the JBI critical appraisal checklist for quasi-experimental studies and non-randomized component of mixed methods studies.

References	Q1	Q2	Q3	Q4	Q5	Q6	Q7	Q8	Q9	Total
3. Angwenyi et al. (54)^*^	Y	NA	Y	N	Y	Y	Y	Y	Y	7/9
5. Fletcher et al. (56)^*^	Y	NA	Y	N	Y	Y	Y	Y	Y	7/9
13. a Rayburn and Coatsworth (39)^*^	Y	NA	Y	N	Y	Y	Y	Y	Y	7/9
16. Solberg et al. (46)	Y	Y	Y	Y	Y	Y	Y	Y	Y	9/9
17. Tandon et al. (49)	Y	Y	Y	N	Y	Y	Y	Y	Y	8/9
18. Thorne and Arnardottir (45)	Y	Y	Y	N	Y	Y	Y	Y	Y	8/9

Y, yes; N, no; NA, not applicable; U, unclear.

^*^Mixed methods studies, where two different appraisal tools have been used.

(1) Is it clear in the study what is the “cause” and what is the ”effect” (i.e. there is no confusion about which variable comes first)? (2) Were the participants included in any comparisons similar? (3) Were the participants included in any comparisons receiving similar treatment/care, other than the exposure or intervention of interest? (4) Was there a control group? (5) Were there multiple measurements of the outcome both pre and post the intervention/ exposure? (6) Was follow up complete and if not, were differences between groups in terms of their follow up adequately described and analyzed? (7) Were the outcomes of participants included in any comparisons measured in the same way? (8) Were outcomes measured in a reliable way? (9) Was appropriate statistical analysis used?.

**Table 3:** Critical appraisal results for included studies using the JBI critical appraisal checklist for qualitative studies and qualitative components of mixed methods studies.

References	Q1	Q2	Q3	Q4	Q5	Q6	Q7	Q8	Q9	Q10	Total
2. Andersson et al. (53)	Y	Y	Y	Y	Y	U	U	Y	Y	Y	8/10
3. Angwenyi et al. (54)^*^	Y	Y	Y	Y	Y	N	Y	Y	Y	Y	9/10
4. Fletcher et al. (55)	Y	Y	Y	Y	Y	N	Y	Y	Y	Y	9/10
5. Fletcher et al. (56)^*^	Y	Y	Y	Y	Y	N	Y	Y	Y	Y	9/10
6. Kaner et al. (47)^*^	Y	Y	Y	Y	Y	N	Y	Y	Y	Y	9/10
12. Parry et al. (57)	Y	Y	Y	Y	Y	Y	Y	Y	Y	Y	10/10
13.b Rayburn et al. (40)^*^	Y	Y	Y	Y	Y	N	Y	Y	Y	Y	9/10

Y, yes; N, no; NA, not applicable; U, unclear.

^*^Mixed methods studies, where two different appraisal tools have been used.

(1) Is there congruity between the stated philosophical perspective and the research methodology? (2) Is there congruity between the research methodology and the research question or objectives? (3) Is there congruity between the research methodology and the methods used to collect data? (4) Is there congruity between the research methodology and the representation and analysis of data? (5) Is there congruity between the research methodology and the interpretation of results? (6) Is there a statement locating the researcher culturally or theoretically? (7) Is the influence of the researcher on the research, and vice- versa, addressed? (8) Are participants, and their voices, adequately represented? (9) Is the research ethical according to current criteria or, for recent studies, and is there evidence of ethical approval by an appropriate body? (10) Do the conclusions drawn in the research report flow from the analysis, or interpretation, of the data?.

The critical appraisal of the nine included RCTs (see Table 1) found quality scores ranging from 8 to 12 out of 13 criteria. All studies used true randomization to assign participants to treatment groups, reported comparable groups at baseline, and treated groups identically aside from the intervention of interest. All trials also measured outcomes consistently and reliably across groups, applied appropriate statistical analyses, and employed suitable trial designs, with any deviations from standard RCT procedures appropriately addressed within the analysis. No studies reported blinding of participants or those delivering treatment, which was expected given the nature of the interventions.

The critical appraisal of the three included quasi-experimental studies and the non-randomized component of the three mixed methods studies (see Table 2) found scores ranging between 7 and 9 out of 9 criteria. All studies clearly established the temporal sequence between cause and effect, ensured comparison groups received the same care aside from the intervention, collected outcome measures both pre- and post-intervention, reported follow-up completeness with any group differences appropriately analyzed, measured outcomes consistently and reliably across groups, and used appropriate statistical analyses.

The critical appraisal of the three included qualitative studies and qualitative components of the four mixed methods studies (see Table 3) found scores ranging between 8 and 10 out of 10 criteria. Only one qualitative study explicitly reported the influence of the researcher and their cultural or theoretical position. Limited reflexivity across studies may affect interpretation of findings, as researcher assumptions, professional backgrounds, and cultural perspectives can shape data collection, analysis, and interpretation within qualitative research.

### Characteristics of included studies

Further detail on the characteristics of the included studies is presented in Appendix 4. The 19 included papers represented 18 unique studies. In line with the review aim, all studies evaluated community-accessible interventions designed to promote positive mental health or prevent poor mental health among fathers during the perinatal period, including psychosocial, psychoeducational, peer support, and digital or technology-assisted approaches delivered to fathers individually, within couples, or in group settings.

Study designs were pre-dominantly quantitative (*n* = 12), comprising nine randomized controlled trials (RCTs), one of which incorporated an embedded qualitative component, and three quasi-experimental studies. Three studies employed mixed methods designs combining quasi-experimental and qualitative data, and three used purely qualitative methodologies.

Studies were conducted across 11 countries, most frequently in Australia (*n* = 5), followed by the United States (*n* = 3) and Singapore (*n* = 2). One study each was conducted in Iceland, Norway, Sweden, the Netherlands, Israel, Iran, Kenya, and Taiwan. The inclusion of studies from 11 countries strengthens the transferability and international relevance of the findings across diverse perinatal care contexts.

Participant characteristics varied considerably across studies and were inconsistently reported. Fathers were typically aged between their late twenties and mid-thirties, although some studies included only fathers aged over 18 or 20 years. Several interventions specifically targeted first-time or expectant fathers, while others included both first-time and experienced fathers or couples transitioning to parenthood. Most studies were conducted in high-income countries and commonly involved married or cohabiting fathers, many of whom were employed and had university-level education. Socioeconomic diversity varied across studies, with some samples including pre-dominantly middle- or higher-income participants, while others included fathers from lower-income households. Ethnic and cultural representation also differed substantially, including pre-dominantly White or Caucasian samples in several Western studies alongside more ethnically diverse populations in studies conducted in Kenya, Singapore, Taiwan, Iran, and the United States. However, reporting of participant demographics was inconsistent across studies, limiting detailed comparison of how interventions may operate across different socioeconomic, cultural, and family contexts.

Interpretation of paternal-focused outcomes should be approached cautiously, as half of the interventions were couple-based (*n* = 9) and half were father-specific (*n* = 9). In some studies, father-specific effects were difficult to separate fully from broader couple or family outcomes, potentially influencing the extent to which observed benefits can be attributed specifically to fathers' participation. In terms of timing, six studies delivered interventions during the antenatal period, two focused on the postnatal period, and the remaining ten spanned the broader perinatal period (pregnancy to 12 months postpartum).

Intervention delivery formats also varied. Six programmes were delivered through group sessions (including mindfulness, antenatal education, new father support groups, and counseling), three used home visiting models, three were text-message based and one was telephone based. The remaining five interventions used mixed delivery modes, including: 1) group sessions plus telephone support, 2) mobile app plus peer support, 3) mobile app plus telephone support, 4) telephone support plus text, and 5) home visiting combined with booklet, video and telephone support.

Collectively, these interventions reflected a broad range of community-based approaches delivered across primary care and home-visiting services, voluntary-sector and peer-support programmes, group-based community settings, and digitally self-accessed or technology-assisted formats. This diversity highlights the potential flexibility of community-based paternal mental health support across different service contexts and levels of accessibility.

### Findings of the review

The primary outcome domains assessed across studies included mental wellbeing, prevention of poor mental health, stress reduction, psychosocial adjustment, coping, parenting confidence, relationship quality, and social connectedness. Secondary outcomes commonly included depression, anxiety, perceived stress, and self-efficacy.

Findings from the quantitative evidence on the effectiveness of community-based interventions to promote mental wellbeing or prevent poor mental health among men in the perinatal period were mixed. Only a small number of studies demonstrated statistically significant improvements in fathers' mental health outcomes, and these effects were often selective rather than consistent across all measures. For example, Ammerman et al. (41) reported significantly lower depression scores, reduced PTSD symptoms, and lower parenting-related personal distress among fathers in the intervention group. Similarly, Li et al. (42) and Mohammadpour et al. (43) found significant reductions in post-birth or state anxiety following intervention, but no effects on trait anxiety or perceived stress. More comprehensive benefits were reported by Shorey et al. (44), who observed significant improvements in depression, anxiety, parenting self-efficacy, parental bonding, perceived social support, and parental satisfaction at 1 and 3 months postnatally, and by Thorne and Arnardottir (45), who found significant improvements across depression, anxiety, self-esteem, and dyadic adjustment. Overall, however, many interventions showed modest or limited effects, highlighting variability in effectiveness across outcomes and studies.

Home-visiting interventions showed the most promising, but still inconsistent, evidence. The Family Foundations programme demonstrated significant benefits for paternal emotional wellbeing, including reduced trauma-related distress, improved coping with the transition to parenthood, and strengthened father–infant interactions (41). Similarly, an earlier home-visiting programme reported clinically meaningful improvements in fathers' depression, anxiety, self-esteem, and dyadic adjustment, with 25% of participants showing substantial reductions in depressive symptoms (45). In contrast, a more recent public health nurse–led home-visiting programme found no significant impact on fathers' depressive symptoms at 6 weeks or 3 months postpartum, indicating no clear benefit over usual care (46).

Group-based randomized controlled trials generally produced modest or short-term effects. Some studies reported reductions in fathers' state anxiety (42, 43) or improvements in relationship quality and confidence in supporting partners (47, 48). However, these benefits were typically not sustained and did not extend to broader outcomes such as depressive symptoms, trait anxiety, or perceived stress. A cognitive-behavioral therapy (CBT)-based psychosocial intervention demonstrated moderate, statistically significant reductions in perceived stress at 3 and 6 months postpartum (*d* = 0.48–0.66), but showed no significant effects on depressive symptoms, anxiety, or perceived partner support (49).

Multi-component and psychoeducational interventions generally showed no significant advantages over usual care in improving paternal mental health outcomes (50, 51). Digital interventions yielded variable findings. One structured telephone- and app-based programme significantly improved fathers' self-efficacy, bonding, and perceived social support, alongside reductions in postnatal depression and anxiety (44). However, a subsequent app-based intervention incorporating peer support demonstrated limited effectiveness across mental health outcomes (52).

Even where quantitative studies did not demonstrate statistically significant improvements in mental health outcomes, qualitative data from mixed-methods studies and standalone qualitative studies consistently reported positive perceived impacts. In total, 39 qualitative findings (Appendix 2) and 11 qualitized findings (Appendix 3) were synthesized into the following integrated findings:

1) Reduced social isolation and increased feelings of connection and belonging.
2) Validation, emotional reassurance, and access to safe, non-judgmental spaces.
3) Strengthened paternal identity, confidence, and readiness for fatherhood.
4) Enhanced father–infant bonding and caregiving involvement.
5) Improved partner relationships and co-parenting through better communication and confidence.
6) Increased knowledge, coping strategies, and mental health literacy.

**1) Reduced social isolation and increased feelings of connection and belonging**

**Figure 2:**
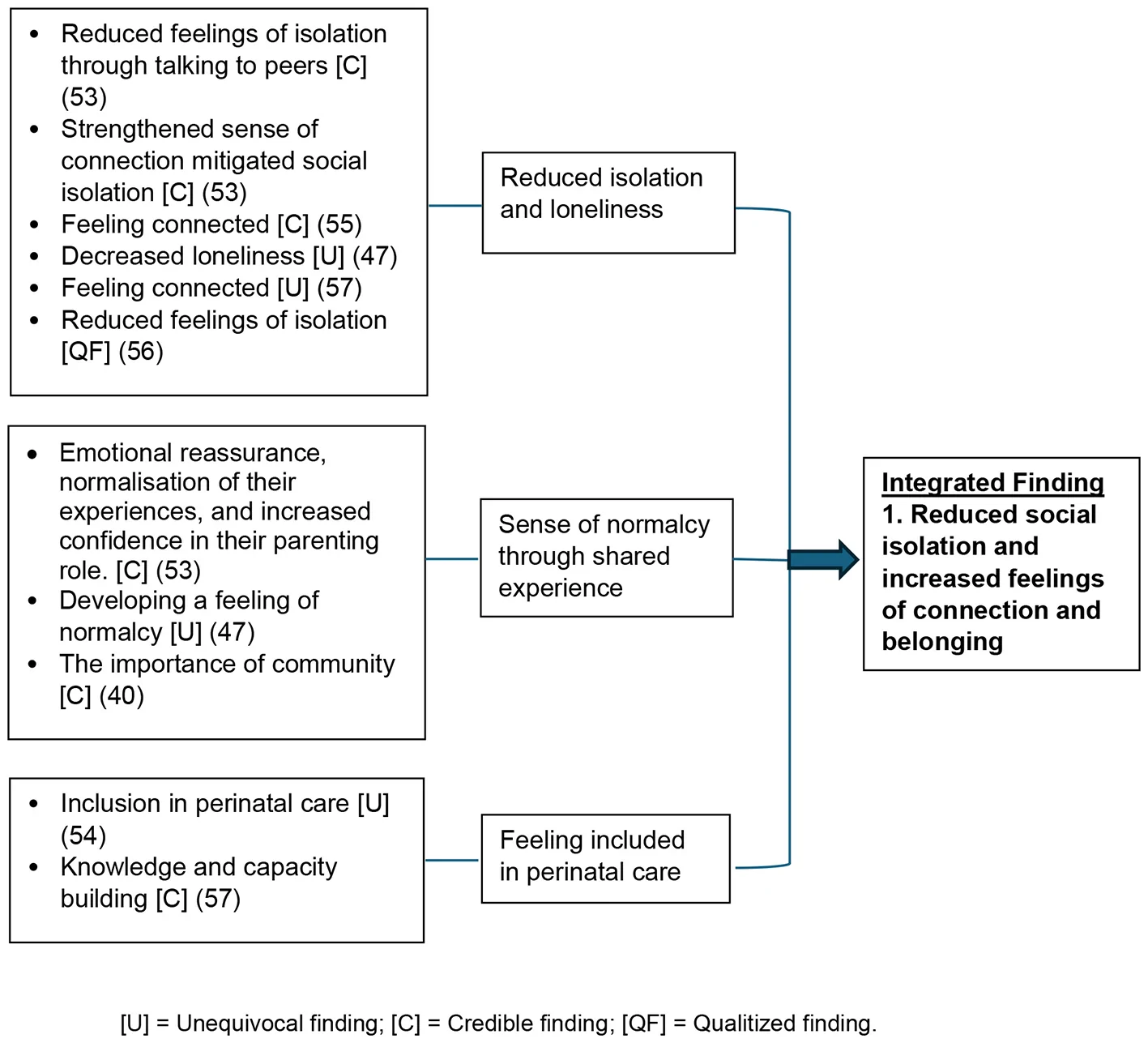
Integrated finding 1-Reduced social isolation and increased feelings of connection and belonging.

A total of 10 qualitative findings (four unequivocal, six credible) and one qualitized finding from seven studies (39, 47, 53–57) generated three categories that together formed the first integrated finding (see Figure 2).

Across multiple interventions, fathers reported that peer support, father-only groups, and digitally mediated interventions (e.g., SMS and telephone-based programmes) helped counter loneliness, foster a sense of normalcy, and create meaningful connections with others undergoing similar transitions (47, 53, 55, 56). As one father described,

“*It felt good that it was called support and not help. It felt more like, here is a person who went through something similar”* (53).

Another reflected on the reassurance provided by digital contact:

“*Sometimes you're looking for support, and you're looking for someone just to get in touch with you and just be like, ‘Hey, how are ya?' …even if it's not a friend, at least it's something that's reminding me that I'm doing a good job”* (55).

This sense of connection was further reinforced in interventions specifically designed for fathers. The qualitized finding was formed from Fletcher et al.'s (56) study where 87.5% father felt the text messages helped lessen their sense of isolation. Feeling included in perinatal care and recognized in their role as fathers also strengthened belonging and engagement as highlighted below. One participant noted,

“*…what I liked was that even us men were included in the care of our children, because you see most of the times men are not included in [programmes], it is only women…”* (54).

Similarly, another father highlighted how father-focused group programmes facilitated social connection:

“*[The program staff member] puts you in the middle [of the group] and talks about what's around…how you connect and talk to others…other dads. You feel connected …I talk to other dads there and that wouldn't happen without this program”* (57).

These opportunities for connection provided emotional reassurance, normalized fathers' experiences, and increased confidence in their parenting role (53). Fathers recognized that the challenges they faced were shared by others, enhancing a sense of normalcy:

“*You see [in group meetings] that others go through similar things…You really see it, and then you say: ‘Yes, I'm normal, I'm average, I'm okay”* (47).

Father-only interventions, in particular, emphasized the value of belonging to a supportive peer network: “*Community with other fathers is fantastic”* (39). Together, these experiences contributed to fathers feeling less alone, more supported, and more confident as they navigated the transition to fatherhood. These relational benefits were reported even when quantitative outcomes showed limited changes in depression or anxiety, highlighting social connection as an important mechanism for promoting paternal wellbeing. newline

**2) Validation, emotional reassurance, and access to safe, non-judgemental spaces**

**Figure 3:**
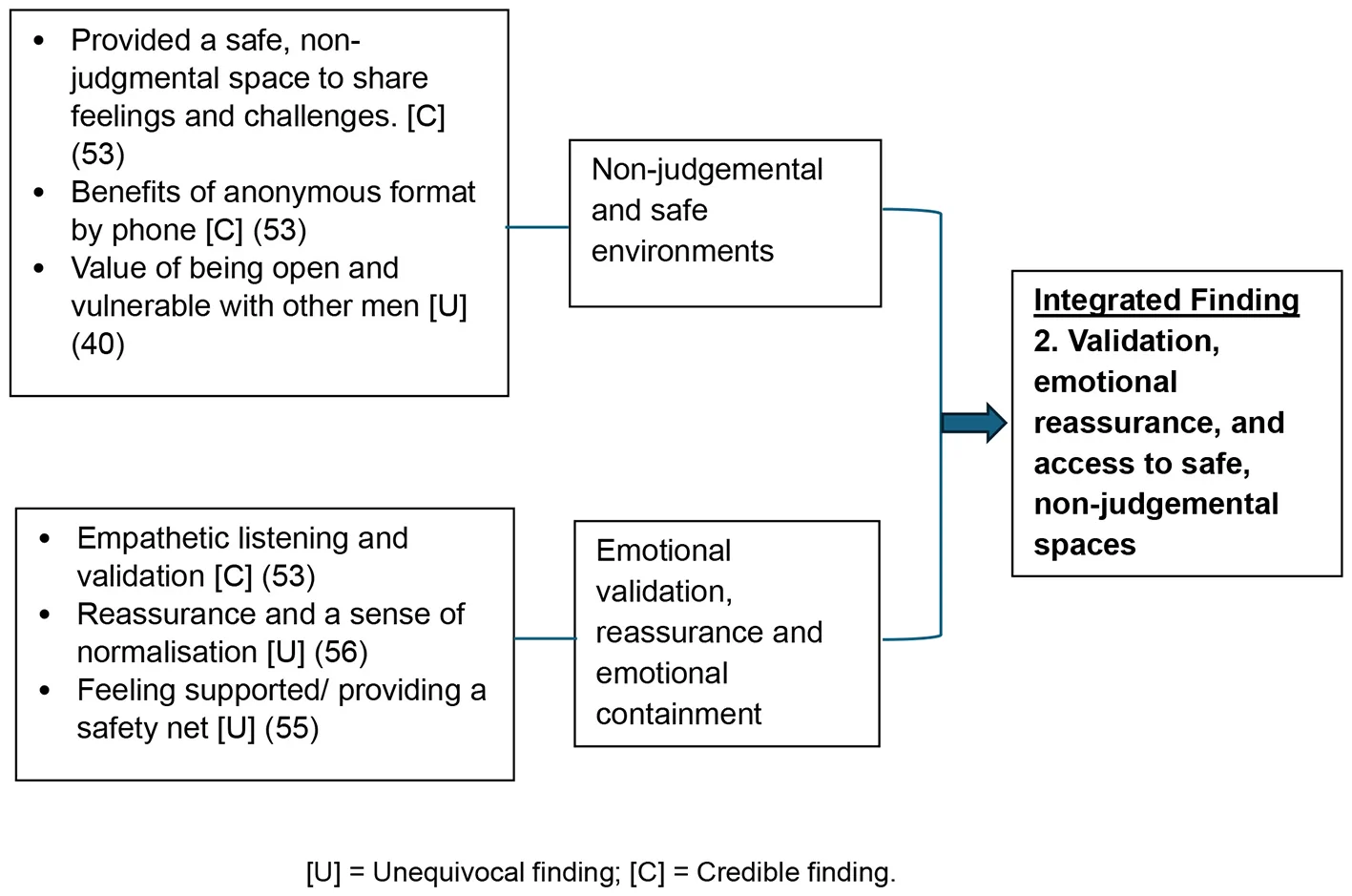
Integrated finding 2-Validation, emotional reassurance, and access to safe, non-judgemental spaces.

A total of six qualitative findings (three unequivocal, three credible) from four qualitative studies (40, 53, 55, 56) contributed to the two categories underpinning the second synthesized finding (see Figure 3). Fathers consistently valued opportunities to share experiences openly, feel listened to, and have their emotions acknowledged without judgement, particularly within peer support, father-only groups, and anonymous or flexible intervention formats (40, 53, 55). For some fathers, anonymity reduced perceived judgement and lowered barriers to participation, with one participant noting that:

“*not seeing each other… became a smaller threshold, because it can be judgmental on appearance”* (53).

Others emphasized the importance of openness and the chance to “*share and learn from others' experiences”* within supportive group environments (40).

Interventions that provided reassurance and normalized fathers' emotional responses were reported to reduce uncertainty and emotional distress during the transition to parenthood. Fathers described feeling reassured by learning that their concerns were common, for example when messages helped normalize infant development:

“*it was reassuring to know that most babies do things in their own time… a good reminder that we weren't alone”* (56).

Others highlighted the emotional relief gained through regular supportive contact, describing how the intervention

“*really helped, particularly with the mental side of everything”*

and served to

“*relieve tension from your day”* (55).

Collectively, these findings indicate that emotionally safe, non-judgemental environments play a key role in supporting fathers' perinatal wellbeing beyond symptom reduction. newline

**3) Strengthened paternal identity, confidence, and readiness for fatherhood**

**Figure 4:**
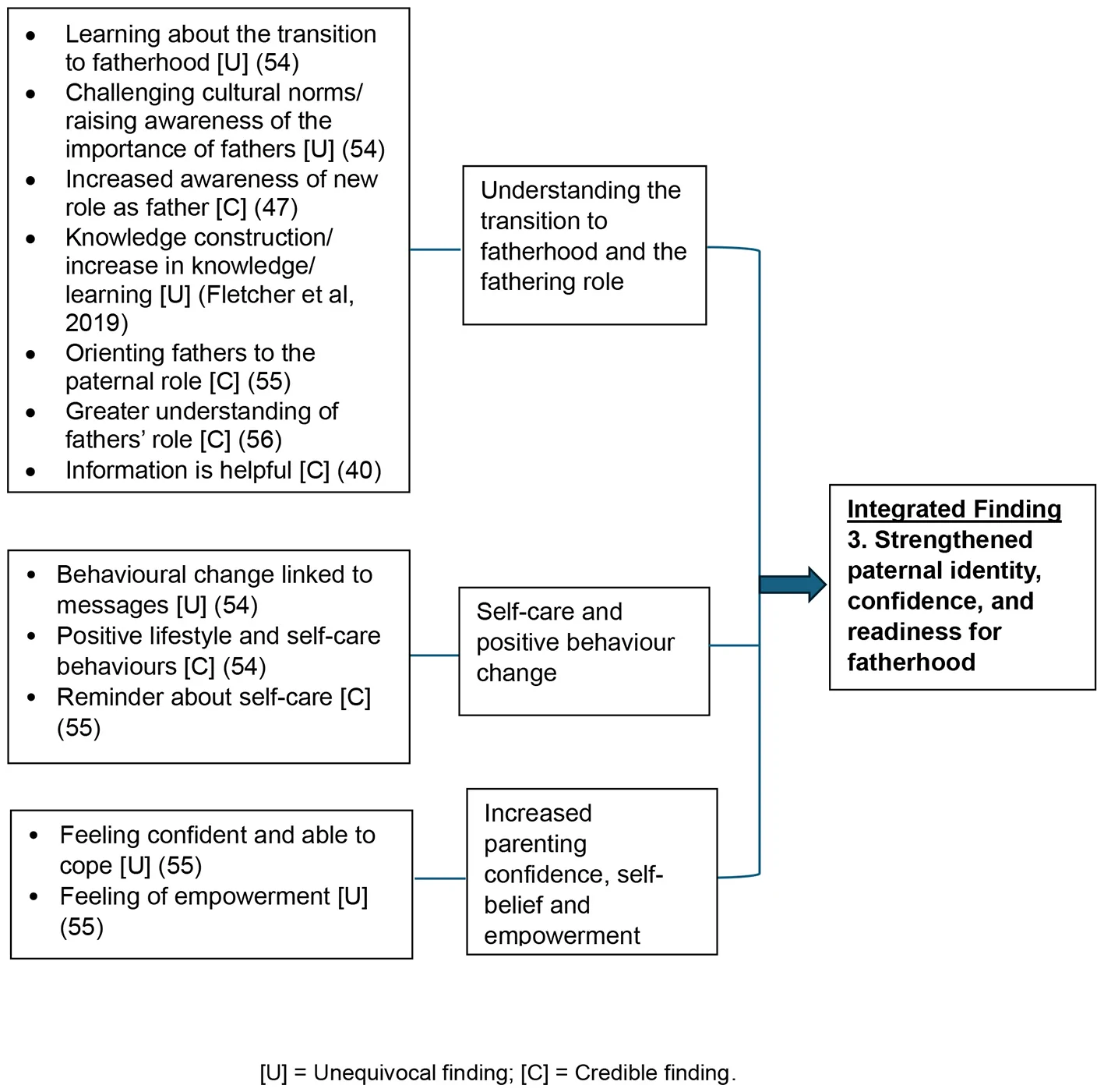
Integrated finding 3-Strengthened paternal identity, confidence, and readiness for fatherhood.

Across five studies (40, 47, 54–56) 12 qualitative findings (six unequivocal, six credible) were identified, forming two categories that together contributed to this synthesized finding (see Figure 4). Fathers consistently reported that educational, peer-based, and digitally delivered interventions supported the development of paternal identity by clarifying their role as fathers, increasing confidence in their parenting abilities, and enhancing preparedness for the transition to parenthood (54–57). Interventions that explicitly acknowledged fathers and positioned them as active and valued caregivers strengthened fathers' emotional connection with their infants and reinforced a sense of importance and purpose, as reflected by one participant:

“*…they helped make more of a connection with her than I otherwise would have as they were worded from the baby and made me feel important and that my role was important to her”* (56).

Beyond attitudinal change, fathers also described tangible behavioral changes that reinforced their identity and confidence as caregivers. Digitally delivered messages prompted shifts in everyday behaviors, including increased engagement in household tasks, healthier coping choices, and greater attention to self-care, which fathers perceived as integral to being a capable and present parent (54, 55). One father explained how behavior change became visible to his partner:

“*The messages changed my behavior… she noticed there is a way these messages were helping me to change”* (54).

Such changes were associated with improved self-regulation, wellbeing, and relational dynamics, further supporting fathers' readiness for parenthood.

Across studies, gaining knowledge about fatherhood, understanding expectations, and receiving encouragement and validation contributed to increased feelings of empowerment, competence, and capability, supporting men's psychological adjustment during both the antenatal and postnatal periods (47, 53). Fathers described a shift from a peripheral or assistive role toward active and meaningful involvement in their child's life, illustrated by one father's reflection:

“*Today, I feel like I'm in a different place, I'm more involved… I can fully participate in my child's growth… I'm not just assisting… I am fully involved”* (47).

These perceived benefits highlight paternal identity, confidence, and role recognition as key dimensions of perinatal mental health promotion that may not be fully captured by symptom-focused outcome measures. newline

**4) Enhanced father–infant bonding and caregiving involvement**

**Figure 5:**
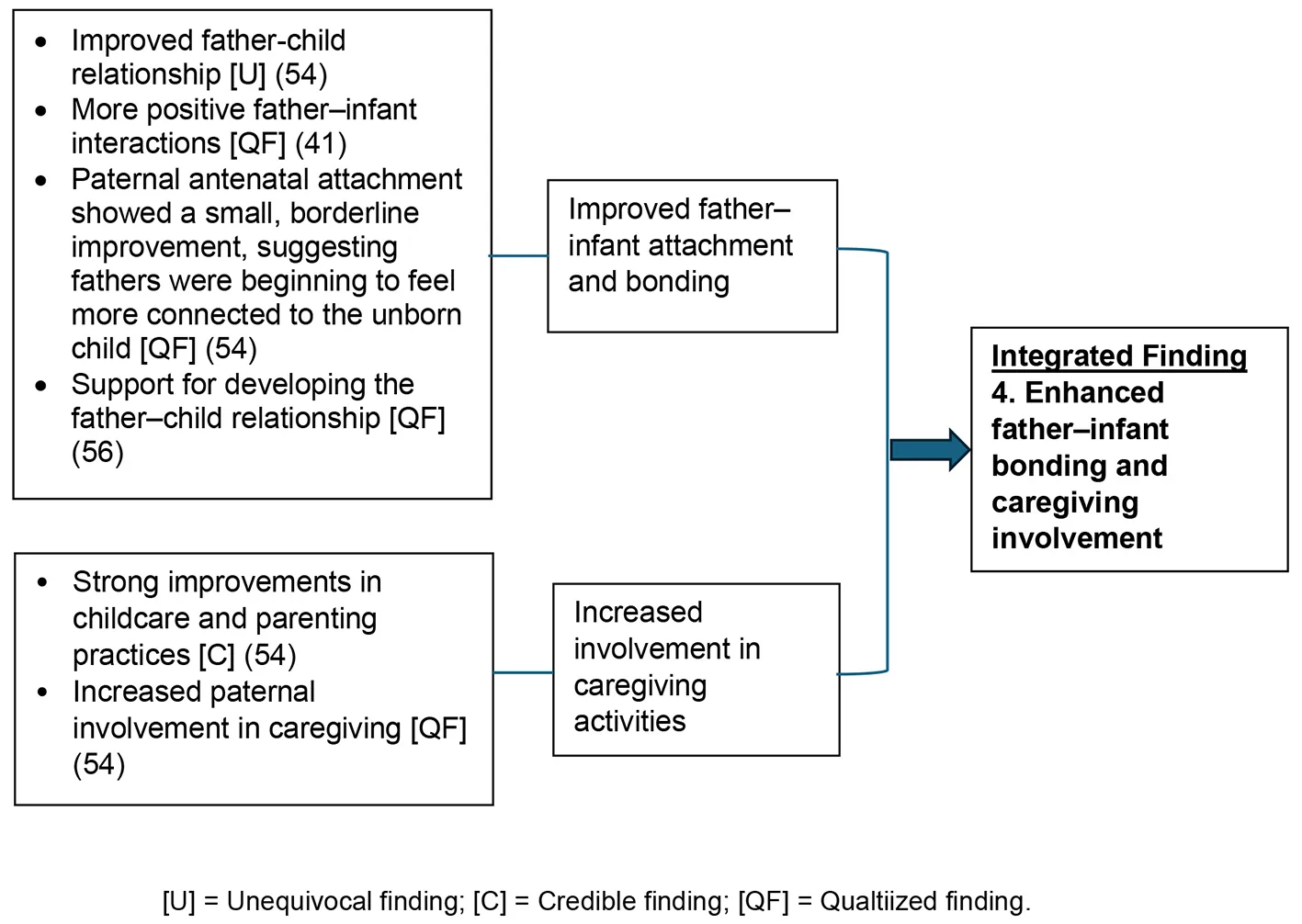
Integrated finding 4-Enhanced father–infant bonding and caregiving involvement.

A total of two qualitative findings (one unequivocal, one credible) from one study (54) and four qualitized findings from three studies (41, 54, 56) contributed to the two categories underpinning this integrated finding (see Figure 5).

Across the interventions, fathers described increased engagement in caregiving activities and a stronger emotional connection with their infants. Participation in father-focused programmes, group activities, and digitally mediated support was reported to encourage hands-on involvement, from routine care to interactive play, enhancing both confidence and competence in their parenting role. As one father reflected,

“*The [SMS4baba messages] changed my mind because through playing with the baby I discovered it helps with his brain development… I learnt by playing with the baby…the baby can communicate…if I sing…the baby utters sounds because they are still small but can smile…”* (54).

In the study by Fletcher et al. (56), 80% of fathers agreed that the text messages supported their bond with their child, indicating that fathers felt more informed and confident in building early relationships. Improvements in childcare and parenting practices were also particularly strong, with a very large effect size reported in Angwenyi et al. (54) (Cohen's *d* = 2.17). When qualitized, these quantitative findings reflect substantial gains in fathers' practical skills, confidence, and competence in caring for their child. These changes extended beyond routine caregiving to include developmentally supportive interactions, structured routines, and proactive engagement with the child's needs, highlighting the interventions' role in promoting both practical competence and relational attunement between fathers and their infants.

Collectively, these experiences suggest that targeted support can enhance both the practical and emotional dimensions of fathering, promoting early bonding and sustained caregiving involvement. newline

**5) Improved partner relationship and co-parenting through better communication and confidence**

**Figure 6:**
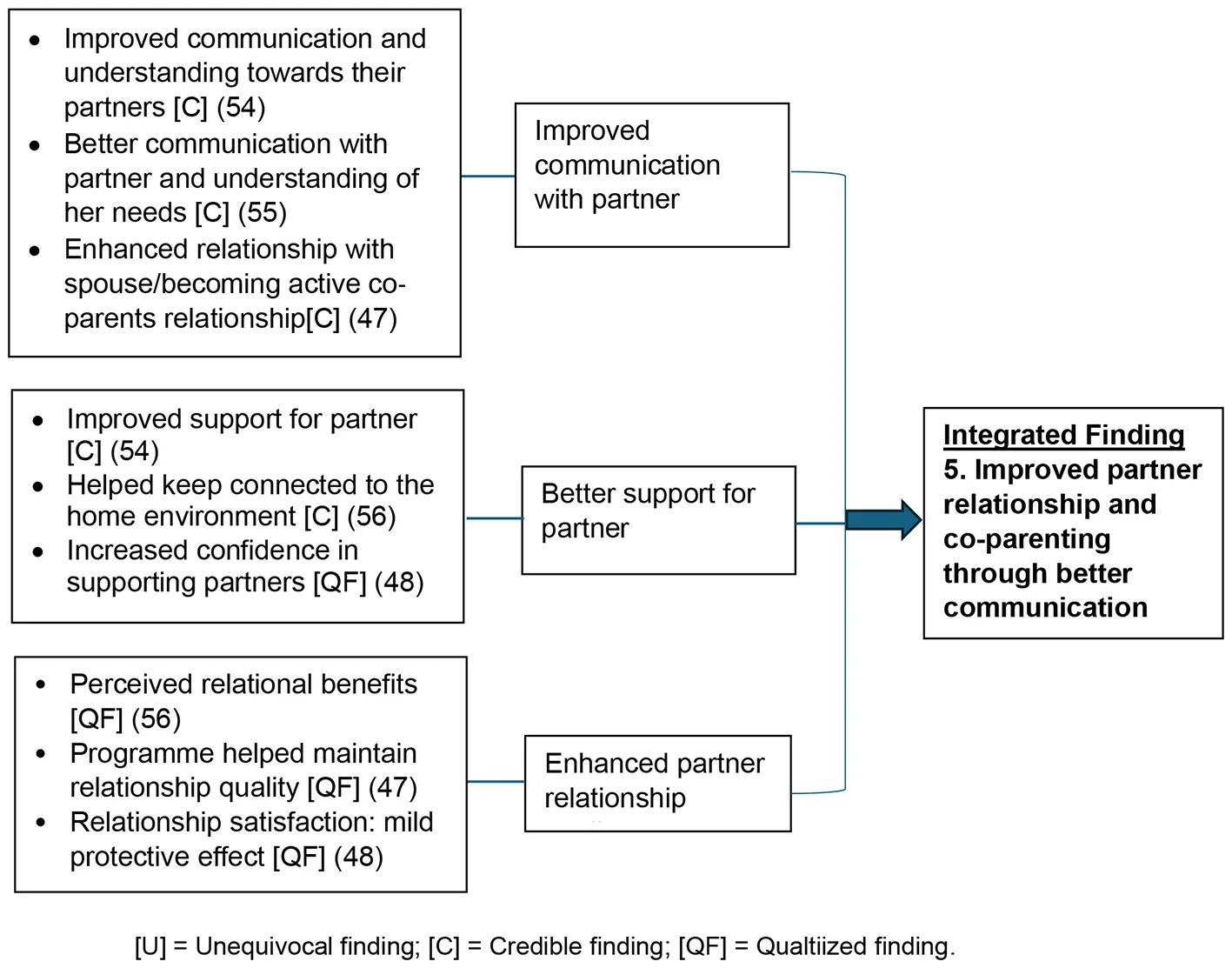
Integrated finding 5-Improved partner relationship and co-parenting through better communication.

Across five studies, five credible qualitative findings and four qualitized findings converged to show that father-focused interventions strengthened partner relationships and co-parenting by increasing fathers' confidence in supporting mothers, improving couple communication, and protecting relationship quality during the transition to parenthood (47, 48, 54–56) (See Figure 6).

Qualitative evidence showed that fathers gained confidence and motivation to actively support their partners, particularly in caregiving and domestic tasks. One father described a shift in mindset:

“*I used to think we leave all the work for women to do… I came to learn I need to help… and saw this was a good thing”* (54).

This was supported by qualitized data from Baby Steps Wellbeing online group programme, which demonstrated a small but significant increase in fathers' confidence to support their partners (Cohen's *d* ≈ 0.26; 46).

Interventions encouraged open discussion and shared understanding of maternal experiences. Fathers reported greater involvement in antenatal care and enhanced unity with their partners:

“*I learnt a lot and became informed that the mother, father, and baby are one… we are more united”* (54).

Other fathers noted that digital messages normalized their partners' feelings and reduced conflict:

“[the text messages] *helped in smoothing it out and not causing a lot of conflicts”* (55).

Group-based programmes also enabled men to practice expressing emotions, which translated into more effective couple communication and a more active co-parenting relationship (47).

Quantitative findings indicated that interventions buffered against the typical postpartum decline in relationship quality. While fathers in comparison groups experienced significant decreases, those in intervention groups maintained relationship quality over time, demonstrating a protective effect (47). Similarly, baby steps wellbeing fathers showed a smaller decline in relationship satisfaction than controls, indicating a mild protective effect (48).

Overall, the integrated evidence suggests that these programmes aimed at fathers enhanced co-parenting and partner relationships by fostering confidence in supporting mothers, improving communication, and protecting relationship quality during early parenthood. newline

**6) Increased knowledge, coping strategies, and mental health literacy**

**Figure 7:**
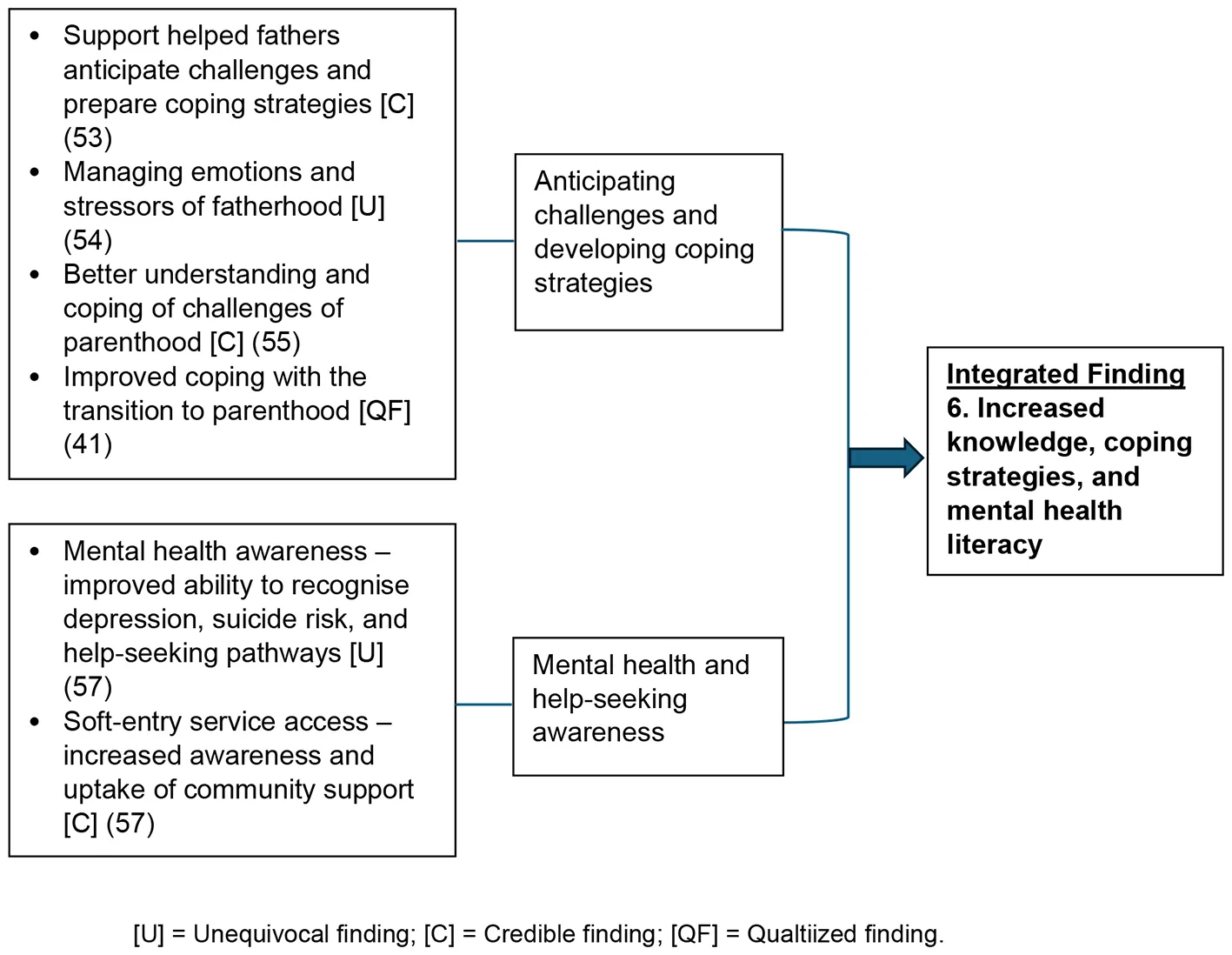
Integrated finding 6-Increased knowledge, coping strategies, and mental health literacy.

Across five studies, three credible and two unequivocal qualitative findings, supported by qualitized evidence, indicate that father-focused interventions enhanced fathers' understanding of the transition to parenthood, strengthened coping strategies for managing stress and emotions, and improved mental health literacy and help-seeking awareness (41, 51, 54, 55, 57) (see Figure 7).

Fathers reported that programme support helped them anticipate common challenges of early parenthood and respond more calmly and confidently. Increased emotional regulation and confidence in the parenting role were evident, with one father noting:

“*I don't care about the baby screaming. I'm calm and that's perfectly fine”* (53).

Similarly, fathers described learning practical strategies to manage anger and stress while ensuring infant safety, such as pausing and calming themselves before responding:

“*If the baby cries a lot and starts to make me feel angry, I put the baby somewhere safe… first calm down and later carry the baby”* (54).

These qualitative accounts align with qualitized evidence demonstrating improved coping with the transition to parenthood among fathers receiving support (41).

Interventions also supported fathers to recognize emotional triggers related to work stress and parenting demands and to adopt adaptive coping responses. Fathers described learning to separate external stressors from family interactions:

“*If angry or have come from work angry, do not carry the anger with you home”* (54),

reflecting increased preparedness to manage the psychological demands of fatherhood.

In addition, fathers gained a clearer understanding of paternal mental health, including depression and suicide risk, challenging assumptions that these concerns primarily affect mothers. Participants reported improved recognition of warning signs and greater awareness of available supports:

“*It really opened my eyes… how I could get help and stuff… all the help that's out there”* (57).

Male-only settings were particularly valued for enabling open discussion and reducing stigma:

“*It was good to talk about these things [depression and suicide] without the women being present”* (57).

Programmes also functioned as “soft-entry” points to community services, increasing fathers' awareness of and engagement with local supports and reducing barriers to help-seeking:

“*I never knew we could get so much help… you don't need referrals either”* (57).

Overall, the integrated evidence suggests that these interventions enhanced men's preparedness for fatherhood by strengthening coping strategies, emotional regulation, and mental health literacy, while also promoting early help-seeking and engagement with community supports.

One qualitative and four qualitized findings (as highlighted in Appendix 2, 3) were not included in the overall integrated findings because they primarily reported engagement, acceptability, and experiential or process-related outcomes, with limited or inconsistent evidence of measurable effects on fathers' mental health. They are however further discussed in the next section.

## Discussion

By integrating quantitative and qualitative evidence, this review addressed an important gap in understanding how community-based preventive and promotional interventions for fathers operate in practice and are experienced by men. Although quantitative evidence for effectiveness on standardized paternal mental health outcomes was mixed, fathers consistently reported meaningful psychosocial and relational benefits from participation.

Quantitative findings demonstrated that relatively few interventions produced statistically significant improvements in anxiety or depressive symptoms, and where effects were observed they were generally modest, consistent with previous reviews highlighting small effect sizes, methodological limitations, and outcome heterogeneity (16, 25, 28). Home-visiting interventions appeared most promising, particularly where fathers were actively engaged, while group-based and psychoeducational approaches demonstrated mainly short-term benefits. Digital interventions produced mixed findings, although interventions incorporating human interaction, tailoring, or structured support appeared more effective than standalone app-based approaches (27). Substantial heterogeneity across intervention design, delivery, timing, and outcome measurement limited comparability between studies and suggests that no single intervention model is universally effective.

Rather than intervention format alone, the findings indicate that effectiveness may depend on mechanisms that address fathers' relational and psychosocial needs. Across intervention types, opportunities for social connection, recognition of fathers' caregiving role, and access to practical and emotional support emerged as key mechanisms underpinning positive experiences. Group-based and peer-support approaches were particularly effective in fostering validation, belonging, and paternal identity development, while father-specific and non-judgemental environments appeared to facilitate emotional openness and reduce isolation. These findings align with literature suggesting that men often prefer relational and informal forms of support and may experience barriers to traditional help-seeking due to stigma and gender norms (58, 59).

The qualitative findings also suggest that paternal wellbeing during the transition to fatherhood is not adequately captured through symptom-focused measures alone. Fathers consistently described improvements in confidence, coping, social connectedness, partner and infant relationships, and paternal identity, despite limited measurable changes in depression or anxiety. These findings suggest that community-based interventions may exert their primary effects through psychosocial, relational, and identity-based pathways rather than direct symptom reduction (14). The discrepancy between quantitative and qualitative findings therefore raises important questions regarding how prevention and mental wellbeing are conceptualized and evaluated within paternal perinatal mental health research.

Several theoretical perspectives help explain these findings. Social support theory proposes that emotional, informational, and peer support can buffer stress during periods of transition (60), while social identity and role adaptation theories emphasize the importance of developing a valued paternal identity and adjusting to new caregiving responsibilities (61, 62). Interventions that explicitly recognized fathers as active caregivers appeared to strengthen confidence, preparedness, and father–infant bonding, suggesting potential benefits for both paternal wellbeing and early child development (2, 3, 5, 8, 63).

The findings also highlight limitations in current outcome measurement approaches. Standardized measures of depression, anxiety, and stress may not capture changes in domains fathers identified as meaningful, including belonging, confidence, relational functioning, and coping. Improvements in mental health literacy, emotional regulation, and help-seeking awareness may represent important preventative outcomes even where short-term symptom change is limited. These findings support calls for broader outcome frameworks incorporating psychosocial, relational, and wellbeing-based measures alongside traditional symptom-focused outcomes, consistent with wider policy emphasis on prevention and early intervention (13, 14). However, preventative effects should be interpreted cautiously, as most studies relied on short follow-up periods and provided limited longitudinal evidence of sustained impact.

A notable finding was the absence of UK-based intervention studies meeting the inclusion criteria, despite national policy commitments to prevention and community-based paternal mental health support (13–15). This likely reflects a broader gap between practice-based innovation and published evaluation research rather than an absence of father-focused provision. For example, the Dad Matters evaluation (64) demonstrates promising practice in engaging fathers and supporting paternal wellbeing but did not meet inclusion criteria due to methodological limitations. Greater investment in rigorous evaluation of UK-based programmes is therefore needed to strengthen the evidence base.

Although excluded from the integrated synthesis, findings relating to engagement and acceptability provide additional insight into implementation. High retention and satisfaction were reported in some studies despite limited changes in standardized mental health outcomes (50, 56), suggesting that acceptability alone may not translate into measurable symptom change. Practical barriers such as work and time constraints affected participation, while emotionally focused components were valued when adequately supported (40, 50). These findings reinforce the importance of designing flexible and engaging interventions that respond to fathers' practical and emotional needs.

### Limitations

The included studies varied substantially in intervention type, delivery mode, outcome measures, and follow-up duration, contributing to considerable heterogeneity and limiting comparability across findings. Reporting quality was also inconsistent, and the relatively small number of mixed-methods studies constrained the depth of evidence integration possible within the review.

Participant demographics, particularly ethnicity, socioeconomic status, and cultural background, were inconsistently reported, limiting exploration of how interventions operate across diverse contexts. Most studies were conducted in high-income countries outside the UK, restricting transferability to UK settings. Marginalized and underserved groups, including younger, migrant, single, and socioeconomically disadvantaged fathers, were also underrepresented, limiting understanding of equity and accessibility.

Only one qualitative study explicitly reported researcher reflexivity, and the process of qualitizing and integrating findings involved an element of interpretive judgement despite following JBI mixed-methods guidance. In addition, although all full-text articles were independently reviewed by two reviewers, only a proportion of title and abstract screening was conducted independently, increasing the possibility that relevant studies may have been missed.

## Conclusions

This mixed-methods systematic review found that community-based interventions for fathers during the perinatal period demonstrate mixed and generally modest effects on standardized mental health outcomes. However, integrating quantitative and qualitative evidence suggests these interventions may meaningfully support paternal wellbeing through improved social connection, coping, paternal identity, and relationships with infants and partners. The findings indicate that paternal wellbeing is best understood as a multidimensional construct encompassing psychosocial, relational, emotional, and parenting-related domains rather than symptom reduction alone. Community-based interventions appear to support wellbeing primarily through psychosocial and identity-based pathways, highlighting limitations in relying solely on depression- and anxiety-focused outcome measures.

Although evidence for longer-term prevention remains limited, the findings support broader conceptual and measurement frameworks for paternal perinatal mental health and align with policy emphasis on preventive, community-based, and gender-responsive approaches to men's mental health (15).

### Practice recommendations

Health visitors, community practitioners, and perinatal services should adopt father-inclusive approaches that explicitly recognize men as active caregivers and attend to fathers' emotional wellbeing, identity, and support needs during the transition to parenthood. Earlier engagement of fathers within antenatal and postnatal care may help strengthen support, reduce isolation, and facilitate help-seeking during the transition to fatherhood.

Practice approaches should also reflect the relational and psychosocial mechanisms identified within this review. Interventions that promote peer connection, provide father-specific and non-judgemental spaces, and validate fathers' caregiving role may support paternal wellbeing even where measurable symptom change is limited. Flexible delivery models, including home-visiting, digital, and hybrid approaches, may further improve accessibility and sustained engagement by addressing practical barriers such as work and time constraints.

Services should additionally consider whether current approaches to assessing paternal mental health are meaningful and acceptable to men. Distress may not always be expressed through traditional symptom descriptors such as sadness, but through anger, irritability, withdrawal, or reduced coping capacity. Practitioners therefore require appropriate training and communication strategies to support contextually sensitive and father-inclusive discussions about mental health.

### Policy recommendations

The findings support policy approaches that prioritize preventive, community-based, and gender-responsive support for fathers during the perinatal period. Paternal mental health should be more explicitly recognized within perinatal mental health policy, commissioning guidance, and service specifications, with fathers acknowledged as service users in their own right rather than secondary recipients of family-focused care.

Longer-term investment is needed to support the development and evaluation of community-based father-focused interventions, including peer-support, group-based, and flexible digital or hybrid programmes. Workforce policy should also strengthen practitioner capability to deliver father-inclusive and psychologically informed care.

Policy and service evaluation frameworks should move beyond reliance on symptom-focused outcomes alone and incorporate broader indicators of wellbeing, relational functioning, social connectedness, paternal identity, and prevention-related outcomes, consistent with the multidimensional conceptualization of paternal wellbeing identified in this review.

### Future research recommendations

Future research should prioritize rigorous evaluation of UK-based and practice-led interventions, alongside greater inclusion of underserved and underrepresented father populations to improve equity, accessibility, and cultural relevance. Longitudinal studies are needed to determine whether early psychosocial and relational benefits translate into sustained mental health and prevention outcomes over time.

Further mixed-methods research integrating effectiveness and experiential evidence would strengthen understanding of intervention mechanisms and active components. Improved reporting of intervention content, implementation processes, contextual factors, and researcher reflexivity would additionally enhance transparency, interpretive rigor, and replicability across paternal perinatal mental health research.

## Data Availability

The original contributions presented in the study are included in the article/Supplementary material, further inquiries can be directed to the corresponding author.
