# Supplementary material for: Community-based interventions to promote paternal mental wellbeing and prevent poor mental health during the perinatal period: a mixed methods systematic review
Source: Front Public Health. 2026 Aug 14;14:1823143. doi: 10.3389/fpubh.2026.1823143 (PMC13521841; doi:10.3389/fpubh.2026.1823143)
Supplement: Supplementary file 1 [file Supplementary_file_1.docx]

**Appendix – 1: Search Strategy**

**MEDLINE and CINAHL (ESBO)**

S1: (Father* OR Men* OR Paternal OR Dad*)

S2: (Intervene* OR Prevent* OR Evaluat* OR Therap* OR Counsel* OR "Self-help" OR Support* OR Program* OR Service* OR Education OR Psycho* OR Online OR Mobile OR App OR Web OR Peer Support OR Group)

S3: (Antenatal OR Antepartum OR Pregnan* OR Prenatal OR Perinatal OR Postnatal OR Postpartum OR Perinatal)

S4: (Mental Health OR Stress OR "Mental Disorder" OR Anxiety OR Depression OR Emotion* OR Trauma* OR "Health promotion" OR Prevention)

S5: S1 AND S2 AND S3 AND S4
