# Supplementary material for: Community-based interventions to promote paternal mental wellbeing and prevent poor mental health during the perinatal period: a mixed methods systematic review
Source: Front Public Health. 2026 Aug 14;14:1823143. doi: 10.3389/fpubh.2026.1823143 (PMC13521841; doi:10.3389/fpubh.2026.1823143)
Supplement: Supplementary file 2 [file Supplementary_file_2.docx]

**Appendix 2 – Extracted Qualitative Findings**

**Andersson et al. 2024**

1. Benefits of anonymous format by phone [C]

“Good thing about not seeing each other—it became a smaller threshold, because it can be judgmental on appearance” —Father 6.

2. Reduced feelings of isolation through talking to peers [C]

“It felt good that it was called support and not help. It felt more like, here is a person who went through something similar”—Father 1.

3. Emotional reassurance, normalisation of their experiences, and increased confidence in their parenting role. [C]

“The confirmation in the talk that you are a good father [. . .], I feel much stronger and strengthened as a father now than I did before my conversations with my peer”—Father 3.

4. Empathetic listening and validation [C]

“Here he’s also been very supportive and affirmative in that ‘You’re a good dad, you’re a good man, and you’re good at this.”—Father 3

5. Support helped fathers anticipate challenges and prepare coping strategies. [C]

“I’m becoming more confident in my own [parental] role. I don’t care about the baby screaming. I’m calm and that’s perfectly fine”.—Father 4

6. Provided a safe, non-judgmental space to share feelings and challenges. [C]

“This bit is kind of an advice, but not directly—I appreciated it, the stories”—Fathers 4

7. Strengthened sense of connection mitigated social isolation [C]

“What was nice about having a peer father was that I’m the first in my group of friends to become a parent and automatically didn’t have a natural contact to turn to”.—Father 2

“It [the program being anonymous] was an incredible benefit for the person I was the contact for because they didn’t know me. [. . .] I don’t know anything about the person, have no relatives, no friends with that person, which means that that person can talk about things you might not talk to anyone else about”—Peer 7.

**Angwenyi et al. 2024**

8. Inclusion in perinatal care [U]

“...what I liked was that even us men were included in the care of our children, because you see most of the times men are not included in [programmes] it is only women…” (New father, 26 years, post-intervention)

9. Learning about the transition to fatherhood [C]

“I read all the messages because this was my first time as a parent…so I had the desire to know more what to anticipate as my baby develops. So, I was very keen with the messages.” (New father, 29 years, post-intervention)

“...when I received the first messages they were surprising and from the things I read, I later came to see they were true as the baby was growing. I then realized I will need to continue reading the messages…and so I continued learning…” (New father, 31 years, post-intervention)

10. Behavioural change linked to messages [U]

“I didn’t share with her [partner] but eventually she saw the messages coming to my phone…when I used to share with her it was through actions, and the messages changed my behaviour. She noticed there is a way these messages were helping me to change…and she started having more affection towards me.” (Father, 38 years, post-intervention)

11. Challenging cultural norms/ raising awareness of the importance of fathers [C]

“…in my community…most men have the habit of saying parenting is a responsibility for women…but when you share [SMS4baba messages] with such men, they will move away from such mentality and see that the child also belongs to them…” (Father, 38 years, post-intervention)

12. Improved father-child relationship [U]

“Communicating with the baby in the womb. I used to tell the baby, ‘my baby, when you come I will carry you with joy.’ During that time, I used to talk and touch the mother’s womb…(Father, 40years, post-intervention)

“the [SMS4baba messages] changed my mind because through playing with the baby I discovered it helps with his brain development… I learnt by playing with the baby…the baby can communicate…if I sing…the baby utters sounds

because they are still small but can smile…(Father, 43 years, post-intervention)

13. Improved support for partner [C]

“they [SMS4baba messages] helped change our mind-sets because I used to think we leave all the work for women to do…I came to learn I need to help with work; when she is washing clothes I could take the baby, and if the baby is hungry I help out with feeding the baby and saw this was a good thing…(Father, 30years, post-intervention).

14. Improved communication and understanding towards their partners [C]

“…I used to think clinic matters are for women but when I started receiving the [SMS4baba] messages for the first time, I attended the clinic with my [expectant] partner. I learnt a lot and became informed that the mother, father, and baby are one… and got motivated…we are more united and I don’t worry about escorting her to clinics…(Father, 34years, post-intervention).

15. Managing emotions and stressors of fatherhood [U]

“I learnt if angry or have come from work angry, do not carry the anger with you home…” (New father, 22years, post-intervention)

“I remember the message that was about the baby that read, ‘if the baby cries a lot and starts to make me feel angry, I put the baby somewhere safe I first calm down and later carry the baby’…the message made me know how to stay with the baby when in distress and know how to act.” (New father, 42 years, post-intervention).

16. Positive lifestyle and self-care behaviours [C]

“…One way of exercising is to help out with household chores like fetching water, washing clothes…even helping with cooking. When engaged in house chores I find I am exercising, and this is something I am doing through this [SMS4baba] Programme” (Father, 43years, post-intervention).

**Fletcher et al, 2019**

17. Knowledge construction/ increase in knowledge/ learning [U]

“Just recognising the different cries or that they do have different cries. That actually triggered a whole night of research. And it was really good. Because of that, I actually learned a whole heap of stuff that I didn’t even know about.” (T38GG4)

18. Feeling confident and able to cope [U]

“And I've got to say having, as I said before, like having that one pop up to just remind me that bub’s not like grizzling and crying out of spite for me, it’s just that she needs something…it helps when you get a bit stressed”. (TM9C7D)

“I’d say it's something to help you stay on track. To get through a really tough phase in your life and, you know, interact, be able to get reminders of what the

baby's going through, what your partner's going through and how best to cope with the situation”. (WI7LFQ)

19. Feeling of empowerment [U]

“Like I said before knowledge is power. It’s about empowering dads to take an interest and to arm themselves with that kind of information right, because that’s what is going to help you. If you go in blind and you don’t know anything, you’re much more I feel, you’re much more likely to potentially struggle and to feel that powerless and to not be able to you know help or assist. Whereas every time you read something and you know like I would read it in an SMS. I would read it in an article, I would see it on a dad advice video, I would talk to [my partner] about it. And it just reinforces that information. (YEC00D)

20. Better understanding and coping of challenges of parenthood [C]

“it’s not to do with me being a bad parent or anything like that. You know, this is what a normal kid does.”

21. Better communication with partner and understanding of her needs [C]

“I can show her some of the messages and show her that what she’s feeling and what she’s thinking isn’t abnormal.”

“[the texts] helped in smoothing it out and not causing a lot of conflicts or

avoiding a lot of conflicts that probably could have been there.”

22. Orienting fathers to the paternal role [C]

“And I think messages that really resonated with me, and made me think more about what I've got myself in to is when you've said, one of the messages says, like ‘how will you make time for me, Dad?’, so it's putting me in that role before I'm actually in that role.” (WUGX5S)

“As a father you're trying to balance the baby as well as work and as well as any other factors. So, it, I guess in a good way, it re-orientates you to what’s probably more important.” (92TLSH)

23. Feeling supported/ providing a safety net [U]

“really helped, particularly with the mental side of everything”, to “relieve tension from your day”

“The messages were good just as a bit of a support pillar because a lot of the things I had done with her were sort of done during the fog of, not the fog of war but the fog of fatherhood”. (T38GG4)

24. Reminder about self care [C]

“So I’ll give you one example where I was struggling and I needed a bit of time for myself. I was off to the pub, we have a pub that’s about a 20 metre walk from my apartment, not that I drink too much or anything, but I was just like, “I just need a break and I need to go and have a drink,” and I got a text message immediately saying, something along the lines of, “How many units of alcohol are you drinking? Alcohol isn’t the answer, blah, blah, blah”. (2HPDPL)

25. Feeling connected [C]

“Sometimes you’re looking for support, and you're looking for someone just to get in touch with you and just be like, “Hey, how are ya?” And you get these coming through and you go, well even if it's not a friend, at least it's something that's reminding me that I'm doing a good job. (3L3F7L)

**Fletcher et al, 2020**

26. Helped keep connected to the home environment [C]

“Gives you an awareness of what you need to think about, especially about the babies and alerts you to issues you and your partner may be going though.” (P15)

“I had to go back to work very soon after the baby was born so it was good to know what might be going on at home. I could talk with my wife about what happened at home and see it was going ok with the baby or if she needed more help”. (P3)

27. Reassurance and a sense of normalisation [U]

“I was stressing about the baby’s development and it was reassuring to know that most babies do things in their own time and not to really compare with others. Good reminder that we weren’t alone, and messages were helpful in that they were consistent with what was going on most of the time”. (P40)

28. Greater understanding of fathers’ role [C]

“…they helped make more of a connection with her than I otherwise would have as they were worded from the baby and made me feel important and that my role was important to her”. (PD61)

**Kaner et al, 2024**

29. Developing a feeling of normalcy [U]

‘. . .It was actually seeing that everyone shares the same things. . . difficulties. . . fatherhood. . . We are all in the same boat. . .’ (Ron)

‘You see [in group meetings] that others go through similar things. . . You really see it, and then you say: “Yes, I’m normal, I’m average, I’m okay”. . .’ (Yaron)

30. Decreased loneliness [U]

. . .It’s good to hear that people who are supposedly strangers to me have similar experiences.. .it’s a kind of a support. . . to know that I’m not alone here, and everyone is going through the same thing, enjoying/suffering . . . (Alon)

‘The uniqueness of this group is in the possibility to step out of your own worldview. . . It exposes you to other worlds and different perspectives that allow you to explore and experience a subjective world different from your own. . . We came to the group with a clear intention to escape. . . from our own situation’. (Adam)

31. Increased awareness of new role as father [C]

‘Today, I feel like I’m in a different place, I’m more involved. . . The truth is that even the discussions here made me understand that I’m in a different stage, in a stage where I can fully participate in my child’s growth. . . I’m not just assisting. . . I am fully involved’. (Barak)

‘For me personally, it raised many thoughts about things I wouldn’t have thought about on my own, and it made me sit down and talk about them with my wife and dedicate time instead of just lounging in front of the television and dozing off’. (Golan)

32. Enhanced relationship with spouse/becoming active co‐parents [C]

‘. . . The meetings made me more conscious about things that I might not think of daily and just do automatically. . . It encouraged discussions with my partner. . . It was a very positive experience for me, and I really enjoyed it. . .’. (Yaron)

“. . . The significant advantage of this [program] is learning to talk with my partner, who always wants to hear your feelings, and you don’t always manage to express what you feel in the right way (sic). The ability to express these feelings first in a group that understands and accepts the male perspective was very helpful in personal conversations with her. For me, it was the most significant advantage.”(Michael).

**Parry et al 2019**

33. Knowledge and capacity building– increased parenting confidence and understanding [C]

“We talked about all sorts of things…skin to skin contact for dad and baby, looking into baby’s eyes, breast feeding. It’s very good for us dads so we know, we can help. So much information it was really good. I felt comfortable asking questions. I feel I know a lot more now…you know what to expect”. (D2)

34. Mental health awareness – improved ability to recognise depression, suicide risk, and help-seeking pathways [U]

“You don’t think about it much in dads. You think about it in the mums all the hormones and everything. It really opened my eyes and gave me lots to think about, you know how I could get help and stuff. They talk about that too all the help that’s out there”. (D1)

“We went through male depression…I was surprised that men get it too-… extra information about where to go and to help us cope. All the information on depression, suicide in fathers all helps and the information on baby and mum helps me worry less. It was good to talk about these things [depression and suicide] without the women being present”. (D4)

“We need to know where to go to get help [for mental health issues] the Antenatal Dads stuff lets you know what to do and where to get help. You can bring stuff out in the open…get help early. Yes, the early warning signs… who to contact and that”. (D2)

“We’ve had four suicides here recently…all within six months and I knew them all so I already feel down in the dumps… we talked about going to the doctor and stuff or talk to someone. I’ve seen it with them they all hold it in…it has a roll on effect… having more knowledge and knowing what to expect helps you deal with it [depression]”. (D5)

35. Soft-entry service access – increased awareness and uptake of community support [C]

“You know, I came here and I didn’t realise just how many services we have in Murray Bridge. I never knew we could get so much help. You don’t need to go anyway to get a referral you can attend here it much easier … the other services you can go to don’t need referrals either. Referrals are such a hassle when you’ve got work, home and everything”. (D3)

36. Feeling connected [U]

“I know to talk to baby, you know, while it’s in my wife’s tummy…I didn’t know that was important …it has helped me feel connected. I didn’t feel that before…I didn’t know about what was happening inside my wife and how the baby can hear. I know what to do now. You can read to baby right from the beginning you don’t need to wait”. (D5)

“[the program staff member] puts you in the middle [of the group] and talks about what’s around…how you connect and talk to others…other dads. You feel connected …I talk to other dads there and that wouldn’t happen without this program”. (D3)

**Rayburn et al, 2021**

37. The importance of community [C]

“Community with other fathers is fantastic.”

“It was great to meet other dads and soon to be dads to share my experience,”

38. Value of being open and vulnerable with other men [U]

“openness” and the “opportunity to share and learn from others’ experiences.”

“hearing personal stories of others’ babies and things I had no idea about.”

39. Information is helpful [C]

“add some excellent skills and resources to the new fatherhood toolkit.”

40. Uncertain about mindfulness [C]

Fathers overwhelmingly liked the reflective listening exercise, perhaps because it paired mindful listening with the experience of being open and vulnerable with another father. One participant said of the program that he “appreciated the emotional and mindful component.” Others expressed wanting more information on how to be successful at certain practices. One said he “could have used more information,” about how to not get caught up in his thoughts, and another said he felt the reflective listening exercise “could have gone more in depth on how.”

Qualitative findings not incorporated into the final synthesised findings.
