# Supplementary material for: Community-based interventions to promote paternal mental wellbeing and prevent poor mental health during the perinatal period: a mixed methods systematic review
Source: Front Public Health. 2026 Aug 14;14:1823143. doi: 10.3389/fpubh.2026.1823143 (PMC13521841; doi:10.3389/fpubh.2026.1823143)
Supplement: Supplementary file 3 [file Supplementary_file_3.docx]

**Appendix 3 – Qualitized Quantitative Findings**

**Ammerman et al. 2022**

1. Enhanced paternal emotional wellbeing among fathers in the FFHV

Estimate = −9.73 (SE = 2.49), p < .01. Indicates FFHV promoted better mental health by reducing depressive symptoms. Quantitative reductions in depressive symptoms among fathers in the FFHV programme (significantly lower scores than controls).

2. Reduced trauma-related distress

Estimate = −27.02 (SE = 7.84), p < .01. Suggests FFHV was effective at preventing or reducing trauma-related distress in the perinatal period.

3. Improved coping with the transition to parenthood

Fathers reported significantly lower personal distress linked to parenting. Estimate = −12.64 (SE = 4.34), p < .01.

4. More positive father–infant interactions

Fathers in FFHV (compliers) were less negatively affective and less intrusive during play interactions with their infants, indicating improved emotional regulation and parenting quality.

**Angwenyi et al 2024**

5. Stable levels of general anxiety and depression: fathers maintained their baseline mental health levels despite the challenges of the perinatal period.

Generalized Anxiety Disorder (GAD-7): No significant pre-post change. Depression (PHQ-9): No significant pre-post change

6. Paternal antenatal attachment showed a small, borderline improvement, suggesting fathers were beginning to feel more connected to the unborn child.

Paternal Antenatal Attachment: Improved slightly, but not statistically significant (Cohen’s d = 0.33, p=0.053).

7. Increased paternal involvement in caregiving

Paternal involvement increased significantly (Cohen’s d = 0.5).

8. Strong improvements in childcare and parenting practices

Childcare & parenting practices ↑ strongly (Cohen’s d = 2.17). The very large effect size for childcare and parenting practices indicates substantial growth in fathers’ practical skills, confidence, and competence in caring for their child.

**Fletcher et al 2020**

9. Strong engagement and sustained participation

The high retention rate (87%) indicates that fathers stayed engaged with the programme and were willing to continue throughout its duration. This reflects a theme of consistent commitment and ongoing involvement.

10. Perceived relational benefits

100% fathers agreed or strongly agreed that the messages helped their relationship with partner. Qualitatively, this suggests fathers experienced the content as meaningful, supportive, and relevant to strengthening their partnerships.

11. Reduced feelings of isolation

87.5% father felt the messages helped lessen their sense of isolation.

12. Support for developing the father–child relationship

A large majority (80%) agreed that the messages supported their bond with their child, indicating that fathers felt more informed and confident in building early relationships.

13. Moderate perceived usefulness and limited engagement of the Mood Tracker

With 44% finding the Mood Tracker helpful, fathers viewed this feature as somewhat useful but less impactful than other programme components. This reflects a theme of partial but limited engagement with mood-monitoring tools.

**Kaner et al, 2024**

14. Emotional wellbeing largely stable, with limited programme impact on depression

Most fathers (77%) showed no signs of postpartum depression, while a smaller proportion (23%) experienced some symptoms (13.2% mild, 9.8% clinical). Statistical analyses indicated no significant differences between groups before the intervention and no meaningful changes over time.

15. Programme helped maintain relationship quality

Before the intervention, the MANOVA analysis on relationship quality showed no significant difference between the groups, F(3, 118) = 2.04, p > .05. However, after analyzing changes over time (2 × 2 MANOVA analysis – Groups × Time), a significant interaction of Groups × Time was found (F1,120) = 8.19, p < .01). Further examination using Simple Effect analyses revealed that the improvement in the intervention group was not significant, F(1, 120) = 1.97, p > .05, while the comparison group showed a significant decrease, F(1, 120) = 6.93, p < .01, Eta2 = .06. While fathers not receiving the intervention experienced relational decline, those in the programme sustained their relationship quality, indicating a buffering or protective effect.

**Kavanagh et al, 2021**

16. No detectable benefit from the Baby Steps Wellbeing intervention over the standard Baby Care programme - in depressive symptoms, psychological quality of life, parenting self-efficacy or social support.

EPDS: Control group: Baseline mean= 4.5, 3 months = 5, 6 months = 4.

Intervention group: Baseline mean= 4.5–4.6, 3 months = no difference, 6 months = 4. Fathers maintained low and stable depressive symptoms across pregnancy to 6 months, with no statistically significant benefit from the Baby Steps Wellbeing intervention. Psychosocial quality of life: No differential improvement by program type; slight overall increase over time.

Control: Baseline mean= 0.47, 3 months = 0.49–0.50, 6 months = 0.51.

Intervention: Baseline mean= 0.47, 3 months = 0.50-0.51, 6 months = 0.52.

Fathers showed a small but significant overall improvement in psychosocial quality of life from late pregnancy to 6 months postpartum, but the intervention made no added difference beyond normal adjustment over time.

Parenting self-efficacy: Fathers scored lower than mothers on average (Cohen d = –0.17). Minimal change over time; no strong benefit from intervention.

Fathers reported lower perceived social support (Cohen d = –0.22). No significant change due to intervention.

17. Relationship satisfaction: mild protective effect

Relationship satisfaction: Fathers’ satisfaction decreased slightly postpartum (Cohen d ≈ –0.26). Those in Baby Steps Wellbeing experienced less reduction compared to Baby Care fathers (Cohen d ≈ 0.20).

18. Increased confidence in supporting partners

Fathers in Baby Steps Wellbeing showed a small but significant increase in confidence to support their partner (Cohen d ≈ 0.26). Qualitatively, this reflects enhanced confidence and perceived competence in supporting their partner during the transition to parenthood.

**Li et al, 2009**

19. A self-efficacy based birth education programme reduced post-birth state anxiety in fathers

Post-birth state anxiety significantly lower in intervention group vs control (43.00 vs 48.12; p = 0.001). Adjusted ANCOVA confirmed a significant intervention effect on reduced anxiety (F = 3.38, p = 0.001). After birth, fathers in the intervention group experienced significantly lower state anxiety than those in the control group.

**Mihelic et al, 2018**

20. Limited measurable impact on mental health outcomes

The intervention did not significantly change depression, anxiety, stress, parenting confidence, or couple satisfaction compared to care-as-usual.

21. High subjective satisfaction with the Baby Triple P programme

Despite limited objective impact, 83.7% of fathers were satisfied or very satisfied, and the mean rating was 5.24/7. Interventions can positively influence fathers’ experience even if standardized mental health measures do not detect changes.

22. Practical barriers to participation emphasising the importance of flexible, accessible program delivery for fathers in the perinatal period.

Attrition was relatively high (~27%), with fathers citing lack of time and work commitments as main reasons for dropout.

**Missler et al 2020**

23. Limited evidence of the effectiveness of universal prevention program in decreasing parental distress or in increasing caregiving quality

Both groups showed a rise in distress levels and a decrease in well-being from t0 to t3, after which distress levels returned to baseline. There were no significant differences in distress between the intervention and control groups at 6 (t3) or 10 weeks after birth (t4).

At baseline, 4 fathers in the intervention group (8.9%) scored above the cut-off score for depression (9 or more on the EPDS) and 2 fathers in the control group (4.65%).

With regard to anxiety, 13 fathers in the intervention group (28.9%) scored 8 or more on the HADS at baseline and 11 fathers in the control group (25,6%).

Ten weeks after birth (t4), 4 fathers in the intervention group (13.8%) scored above the threshold for depression, and 5 fathers in the control group (13.9%).

A total of 7 fathers in the intervention group (24.1%) scored above the cut-off for anxiety (8 or more on the HADS) compared to 7 fathers in the control group (19.4%).

**Mohammadpour et al 2021**

24. Reduction in short-term state anxiety

Four weeks after the intervention, fathers in the program reported significantly lower state anxiety than controls.

The mean scores of state anxiety in the intervention group decreased significantly 4 weeks after the intervention compared with the control group (MD: -2.4; 95%CI: − 4.7 to − 0.2; p = 0.030).

25. No change in trait anxiety or perceived stress

No significant differences were observed for trait anxiety or perceived stress at the same follow-up. This indicates that the intervention primarily affected situational, acute anxiety rather than broader, enduring stress or personality-linked anxiety tendencies.

Four weeks after the intervention, no significant difference was found between the two groups in terms of trait anxiety (p = 0.472) and perceived stress (p = 0.635).

**Shorey et al 2019**

26. Telephone based AN & PN education & Mobile health app programme improved parental self-efficacy, bonding and perceived social support and parenting satisfaction at 1 month and 3 months post birth.

1 month postnatal:

- Parenting self-efficacy	p < .001	Improved
- Parental bonding	p < .001	Improved
- Perceived social support	p < .001	Improved
- Parenting satisfaction	p < .001	Improved

3 Months Postpartum: Effects remained significant for fathers in all domains:

- Parenting self-efficacy	higher than control	p = .036
- Parental bonding	stronger bonding	p < .001
- Perceived social support	higher than control	p = .015
- Parenting satisfaction	higher than control	p < .001

27. Telephone based AN & PN education & Mobile health app programme reduced postnatal depression and anxiety 1 month and 3 months post birth.

1 month postnatal:

- Postnatal depression	p < .001	Reduced
- Postnatal anxiety	p < .001	Reduced

3 months postpartum:

- Postnatal depression	lower than control	p < .001
- Postnatal anxiety	lower than control	p < .001

**Shorey et al 2023**

28. The SPA intervention showed limited effectiveness, with depression and anxiety outcomes not reaching statistical significance.

Depression (EPDS): Intervention fathers showed a steady improvement in depressive symptoms from baseline to 6 months postpartum, followed by a rise in EPDS scores after the intervention ended (loss of effect). Their mean EPDS remained low (~score of 5 from 4 months onward), which may explain the limited measurable improvement. Control fathers showed a similar depression trajectory to their partners, suggesting mutual partner influence on mood.

Anxiety (STAI): Intervention fathers had slightly lower anxiety levels than control fathers up to 6 months postpartum, but this difference was not statistically significant. Anxiety decreased over time in both groups.

29. No statistical difference for perceived social support, parenting self efficacy and parental satisfaction in intervention group.

Perceived Social Support (PSSP): Intervention group parents (overall) had significantly higher social support than control, but fathers benefited less than mothers, reporting lower perceived support than mothers and lower engagement with peer support features. Fathers preferred informational support rather than emotional peer support, unlike mothers who actively used peer features.

Parenting Self-Efficacy & Satisfaction (PES, WPBL): No meaningful difference between intervention and control for fathers (high baseline scores, ceiling effect). Both intervention and control fathers maintained high self-efficacy and parenting satisfaction across time.

**Solberg et al 2024**

30. New Families home visiting program had no clear impact on depressive symptoms in fathers during the postnatal period.

No significant EPDS score differences were found between the intervention and the control group at six weeks and three months postpartum:

6 weeks: Mean diff 0.22 (-0.73, 1.16), p = .65

3 months: Mean diff 0.08 (-0.88, 1.15), p = .87

**Tandon et al 2021**

31. This cognitive-behavioural therapy (CBT)-based psychosocial intervention reduces perceived stress at 3 months and 6 months postnatally.

Perceived stress significantly reduced (moderate effect):

3 months: d = 0.48, 6 months: d = 0.66, p < .05

32. This cognitive-behavioural therapy (CBT)-based psychosocial intervention did not reduce depressive symptoms, anxiety or perceived partner support.

No statistically significant differences were found for depressive symptoms, anxiety symptoms, or perceived partner support.

Depressive symptoms decreased (small effect, not statistically significant):

d = 0.35 at 3 months; Anxiety reduced slightly (small, non-significant effect): d = 0.20 at 3 months

**Thorne and Arnardottir 2013**

33. Home visiting programme showed to improve depression, anxiety, self-esteem, and dyadic adjustment among fathers.

25% showed clinically meaningful improvement (EDS drop of 4–10 points). Men showed significant improvement across all scales (depression, anxiety, self-esteem, dyadic adjustment).

Quantitative data summarised in findings section.

Qualitized data incorporated in the integrated findings.

Qualitized data not combined to form a category in the integrated findings.
