# Supplementary material for: Community-based interventions to promote paternal mental wellbeing and prevent poor mental health during the perinatal period: a mixed methods systematic review
Source: Front Public Health. 2026 Aug 14;14:1823143. doi: 10.3389/fpubh.2026.1823143 (PMC13521841; doi:10.3389/fpubh.2026.1823143)
Supplement: Supplementary file 4 [file Supplementary_file_4.docx]

**Appendix – 4**

**List of Included Studies**

| **No.** | **Author**  **and year** | **Methodology** | **Method** | **Setting/**  **country** | **Participants** | **Phenomena of**  **interest** | **Data analysis** | **Authors’ conclusions relating to outcome of interest** |
| --- | --- | --- | --- | --- | --- | --- | --- | --- |
| 1. | Ammerman et al 2022 | RCT | Assessments at  pre-intervention and post-intervention. | USA | 150 dyads included fathers; FFHV = 70, HV = 80. | Impact of an adapted Family Foundations for Home Visiting (FFHV) program on parental mental health, parenting stress, and observed parent–child interactions within low-income home visiting families. | Outcomes were analysed using Complier Average Causal Effect (CACE), estimating effects among fathers who attended ≥9 sessions. | FFHV is a promising community-based program that can promote good mental health among fathers by reducing depression, PTSD symptoms, and parenting stress, and by improving observed father–infant interactions. Reported effects reflect benefits for engaged fathers rather than all randomised participants. |
| 2. | Andersson et al 2024 | Feasibility qualitative study | 6 semi-structured interviews of first-time fathers (and 7 of peers) | Sweden | First-time fathers and trained peer mentors | The implementation and satisfaction with a telephone-based peer support program for new fathers. | Content analysis | The findings provide valuable insights into the potential benefits and  challenges of implementing a telephone-based peer support program for first-time fathers, which  could further inform similar interventions. |
| 3. | Angwenyi et al 2024 | Mixed-methods (Quasi-Experimental & qualitative) | Focus group discussions;  pre-post  evaluation | Nairobi, Kenya | Focus groups – 19 fathers, with young men (under 30 years) and older men (above 30 years) who either had an expectant partner or  had a child below 6 months.  72 fathers completed baseline assessment.  Average age - 30.70 (SD = 6.80), ranging  from 21 to 48 years. 23.6% (n = 17)  were expecting their first child. Most of the fathers were Kenyans (n = 66, 91.7%), Christians (n = 68, 94.4%), lived  with their partners 66 (91.7%), were married (n = 66, 91.7%), and had a secondary level of education (n = 43,  59.7%). | Evaluate the effect of the adapted text only  SMS4baba intervention, on paternal and childcare  involvement, parenting, and father wellbeing outcomes  in a Kenyan urban informal settlement. | Qualitative: Focus Group Discussions – thematic analysis with NVivo  Measures:  Paternal involvement (10-item researcher-developed scale).  Childcare & parenting practices (3-item researcher-developed).  Paternal Antenatal Attachment Scale (PAAS).  Mental health: GAD-7 (anxiety), PHQ-9 (depression).  Analysis: Paired t-tests, mixed model regression, effect sizes (Cohen’s d). | Qualitative reports demonstrate SMS4baba was perceived as informative, helped fathers cope with fatherhood challenges, and messages strengthened father-child relations, and support for their partners in taking up responsibilities, which challenged gendered norms. Pre-post evaluation of the SMS4baba intervention demonstrated positive effects on father-child attachment and father involvement in childcare but not for depression/ anxiety scores |
| **4.** | Fletcher et al 2019 | Qualitative process evaluation | Semi-structured telephone interviews conducted after program completion. | Australia; participants drawn nationally through perinatal health services and social media recruitment. | 40 fathers (out of 74 invited; 58% response rate). | Understanding how and why community-based, mobile text message interventions promote men’s positive transition to fatherhood, improve emotional wellbeing, and reduce isolation —mechanisms of impact underpinning mental health support for fathers in the perinatal period. | Deductive–inductive thematic analysis (using NVivo 12) | SMS4dads supports fathers’ transition to parenthood through both program structure (timely, tailored, father-specific messages) and psychological processes (knowledge, confidence, connection). These elements interact to promote emotional wellbeing, role confidence, and engagement. |
| 5. | Fletcher et al 2020 | Mixed-methods feasibility study – Quasi-experimental & qualitative | Quantitative: Enrolment and engagement data (message interaction, link clicks, Mood Tracker responses), and Qualitative: Semi-structured interviews. | Two Australian regional centres. | Total enrolled: 23 partners (primarily fathers).  Interviewed: 16 participants (70%) completed post-program interviews. | Feasibility, acceptability, and potential psychosocial impact of a mobile text intervention that aims to promote mental health and parenting confidence among partners of women with severe perinatal mental illness. | Interview transcripts coded in NVivo 12 using a pre-existing thematic framework; survey responses tallied. | The adapted text-message program was acceptable and feasible for partners of women with SMI. Partners reported greater confidence, awareness, and connectedness, and valued timely, supportive information. Messages appeared to strengthen partner support for mothers and enhance father–infant engagement. |
| 6. | Kaner et al 2024 | Mixed-methods – RCT & qualitative | post-partum depression symptoms (PPD) using the  Edinburgh Post-Natal Depression Scale (EPDS) and spousal relationship  quality using the Israeli Marital Quality Scale (IMQS). Qualitative  data were collected through session transcripts and feedback questionnaires  that were completed two years post- intervention | Israel | 122 participants  split into intervention (n = 62) and comparison groups (n = 60). | Investigated  attendance rates, fathers’ participation experiences, and the effects  on their psychological well-being and marital quality. | The quantitative analysis used conventional bivariate analyses, t-tests, one-way Anova and 2 × 2Manova (groups by times).  The qualitative data were analyzed using Braun and Clarke’s thematic analysis method | Participants reported feeling a sense of normalcy, decreased  loneliness, increased awareness of fatherhood transition, and becoming active co-parents. Marital quality also showed better results for the intervention group. |
| 7. | Kavanagh et al 2021 | Quantitative - RCT | Web-based self-reports using EPDS and psychosocial quality of life scales at baseline, 3 months postnatal and 6 months postnatal. | Brisbane, Australia, recruited from prenatal education classes. | Total recruited couples: 248 Fathers = 248, randomly allocated: Baby Steps Wellbeing group - 124 fathers.  Baby Care (control) group - 124 fathers. | Evaluation of Baby Steps Wellbeing, a self-guided online intervention for perinatal wellbeing, compared with a control condition web-based self-reports. Primary outcome depression and psychosocial quality of life. Secondary outcomes: relationship satisfaction, social support, and self-efficacy for parenting and support. | Linear mixed-effects analyses | EPDS – no significant difference (p > .3)  Psychosocial QoL- p = .002. Slight improvement for all fathers.  Relationship satisfaction: decreased slightly postpartum (Cohen d ≈ –0.26). Those in Baby Steps Wellbeing experienced less reduction compared to Baby Care fathers (Cohen d ≈ 0.20).  Self-efficacy for support provision: Fathers in Baby Steps Wellbeing showed a small but significant increase in confidence to support their partner (Cohen d ≈ 0.26).  Social support: Fathers reported lower perceived social support (Cohen d = –0.22). No significant change due to intervention. |
| **8.** | Li et al 2009 | Quantitative - RCT | Basic personal information, a childbirth expectations  questionnaire, and a Trait Anxiety Inventory at baseline and two hours after birth. | Taiwan – hospital antenatal group – 3-4 per group | 87 fathers (Experimental n=45, Control n=42).  First-time expectant fathers ≥20 years old, able to read/ write/ speak Chinese.  Partners 34–36 weeks gestation, singleton pregnancy, no complications. | A birth education intervention for expectant fathers, based on self-efficacy theory, aiming to reduce anxiety and support fathers during labour and birth. | Descriptive statistics, independent t-tests, chi-square tests, and ANCOVA to adjust for covariates with significance set at p < 0.05, using SPSS 15.0 | A self-efficacy based birth education programme significantly reduces paternal anxiety during labour and birth. |
| **9.** | Mihelic et al 2018 | Quantitative - RCT (2-arm) | Data collected at 3 time points – baseline, 10 weeks postnatal and 6 months postnatal. Using EPDS, DASS, Oxford Happiness Questionnaire, MSRI, Maternal Self-Efficacy Scale, Maternal Infant Responsiveness Instrument. | Brisbane and Ipswich, Queensland, Australia | 112 fathers randomised (Intervention = 57; Care as usual = 55).  First-time fathers, ≥18 years old  Partner between 20–35 weeks’ gestation  In a couple relationship | Community-based intervention (Baby Triple P) aiming to improve paternal perinatal psychosocial wellbeing.  Four 2-hr Baby Triple P group sessions  conducted during pregnancy between the 20th and 40th  weeks of gestation, followed by four individual 30-min telephone  sessions conducted postnatally once the baby was 6 weeks old. | Intention-to-treat linear mixed models, with fixed effects for group, time, and group-by-time interactions to assess intervention effects. | Baby Triple P did not produce significant improvements in paternal mental health, parenting, or relationship outcomes. Lack of effects is likely due to low father engagement, high attrition, time burden, and a sample that was already psychologically well-adjusted at baseline, limiting room for improvement. |
| **10.** | Missler et al 2020 | Quantitative – Cluster RCT | Online assessments at baseline (26 and 34 wk gestation), 34–36 wk gestation, 2 wks, 6 wks  and 10 wks after birth – through an e-mail with a link to a questionnaire. | Netherlands | 138 pregnant women and 96 partners were randomised. Fathers/ partners - 48 to intervention, 48 to control. | Effectiveness of a psycho-educational  intervention for expecting parents to  prevent postpartum parenting stress,  depression and anxiety | Multilevel linear modelling (MLM) with fixed effects for gender and intervention. | No evidence that the universal prevention program was effective in decreasing parental distress or in increasing caregiving quality. However, parents found aspects of the intervention useful. |
| **11.** | Mohammadpour et al 2021 | Quantitative - RCT | Perceived stress and anxiety questionnaires were completed before  and 4 weeks after the intervention. | Ardabil, Iran  Community health care centres | 102 fathers randomised (51 intervention, 51 control).  Spouses of primiparous pregnant women (20–24 wks gestation) | Effect of counselling on fathers’ stress  and anxiety during pregnancy | Independent t-tests, Chi-square/Fisher’s exact tests for baseline comparisons, and ANCOVA to assess post-intervention effects under an intention-to-treat approach (p < 0.05). | The findings indicate that counselling reduced state anxiety in expectant fathers; therefore, this intervention is recommended to be used to reduce fathers’ anxiety. |
| **12.** | Parry et al 2019 | Qualitative study | 9 fathers - face-to-face or phone interviews  7 fathers - focus group | Murraylands region, South Australia. Delivered in community settings. | 16 fathers and 6 staff of a community-based parenting  program with sessions focusing on fatherhood. | Exploration of an antenatal dads program  aimed at fathers to assess the benefits of such interventions. | Inductive Thematic analysis as described  by Patton (2015) and constant comparative  method as described by Glaser and Strauss (1967). | The fathers found that the male-only sessions assisted them by supporting frank discussions on fatherhood. The study also helped identify the advantages of fathers meeting other fathers through attendance in the program, or even other couples in similar situations that helped fathers to feel less lonely regarding their situation. |
| **13.a** | Rayburn & Coatsworth 2021 | Quantitative Pilot single-group pre–post intervention study (non-randomised, non-comparative) using quantitative surveys and reliable change analysis | Pre- and post intervention surveys to assess efficacy in the areas of stress, depressive symptoms, father involvement  attitudes, and mindfulness. | United States (Colorado State University study region) | 19 expectant and new fathers. Participants were White (89.5%). One  identified as both White and Latino, one  as both White and Asian, and one as Latino. Average age 32. Most had a  bachelor’s degree. All were in fulltime  employment. | Pilot study of a 5-week, father-only group programme aimed to improve stress, depressive symptoms, mindfulness, father involvement attitudes. | Paired pre–post comparisons and reliable change analysis to examine changes in stress, depression, mindfulness, and fathering outcomes. | The program shows promising preliminary evidence for reducing paternal stress and depressive symptoms and improving mindfulness, supporting the value of father-specific perinatal support, but requires larger controlled studies. |
| **13.b** | Rayburn et al 2021 | Mixed methods – as above, plus qualitative component | Qualitative component used written open-ended participant reflections | As above | As above | As above | Thematic analysis | Higher order themes emerged of community, openness/vulnerability, utility of information, and the need for more time. Mindfulness skills evoked mixed sentiments. Expectant and new fathers are not currently well supported by available programs, and there is a clear need for additional interventions focused on fathers’ experiences. |
| **14.** | Shorey et al 2019 | Quantitative - RCT | Data were collected  at 4 time points: pregnancy (3rd trimester), 2 days, 1 month, and 3 months  postpartum. Measuring parenting self-efficacy (PSE), parental bonding, perceived social support, parenting  satisfaction, postnatal depression (PND), and anxiety. | Antenatal clinic of a tertiary hospital in Singapore | 236 parents – couples randomly assigned to intervention (n=118) or control group (n=118). Total fathers 118 (59 in each group) | Effectiveness of a technology-based supportive educational parenting program  (SEPP) on parenting outcomes during the perinatal period in couples | Intention-to-treat linear mixed-effects models to compare intervention and control groups over time | The technology-based SEPP is effective in enhancing parental bonding, PSE, perceived social support and parental satisfaction, and in reducing PND and PNA. |
| **15.** | Shorey et al 2023 | RCT | Self-report online questionnaires sent via WhatsApp at baseline (>24 weeks gestation) and at 1, 2, 4, 6, 9, and 12 months postpartum. | Singapore – public health institutions | n = 200 fathers (part of 200 heterosexual couples) | Effectiveness of a Supportive Parenting App on parental outcomes during the perinatal period. | Linear mixed-effects models (intention-to-treat) | The SPA intervention showed limited effectiveness, with most outcomes not reaching statistical significance, potentially due to the impact of the COVID-19 pandemic. Parents in the intervention group perceived greater social support than those in the control group. Mothers generally benefited more than fathers, indicating different support needs by parent gender. |
| **16.** | Solberg et al 2024 | Prospective non-randomised controlled study with a parallel group design. | Data collected at 28 weeks pregnancy, 6 weeks postpartum, 3 months postpartum using self-report questionnaires (EPDS). Intervention compared to standard care. | Oslo, Norway, community child health service | 197 fathers (intervention 121, control 76)  91.9% first-time fathers  85.3% Norwegian/ Nordic, 14.2% other countries  99% married/ cohabiting, 96% employed  82.2% had university education, 82.2% family income > 750,000 NOK | To assess possible differences in level of depressive symptom in fathers receiving the  New Families home visiting program compared with those receiving standard care from the Norwegian Child Health  Service. | Intention-to-treat, with Mann–Whitney U and chi-square tests to compare groups, and a general linear model (GLM) for repeated measures to assess differences in EPDS score changes over time between intervention and control groups. | No significant EPDS score differences were found between the intervention and the control group at six weeks and three months postpartum. This suggests that the intervention had no clear impact on depressive symptoms during this time-period. |
| **17.** | Tandon et al 2021 | Quantitative - Single-group longitudinal pre–post pilot study (no control group). | Quantitative self-report surveys at baseline, 3 months, and 6 months. | United States (community-based home visiting programs) across 9 sites | 30 father–mother dyads (N = 30 fathers)  Mean father age 27.7 years.  37% Black, 30% Hispanic, 27% White, 7% Other.  Biological and non-biological fathers; co-habitation not required.  80% enrolled post-birth; 20% enrolled antenatally. | Fathers and Babies (FAB) – a cognitive-behavioural therapy (CBT)-based psychosocial intervention to improve paternal mental health, reduce stress and depressive symptoms, and strengthen coping and social support. | Paired t-tests with Bonferroni correction and Cohen’s d effect sizes. | Fathers (and mothers) had statistically significant decreases in perceived stress between baseline and both follow-up time points, with moderate effect sizes generated.  No statistically significant differences were found for depressive symptoms, anxiety symptoms, or perceived partner support. Future research needed for  evidence of intervention effectiveness  . |
| **18.** | Thome and Arnardottir 2013 | Quantitative - Single-group quasi-experimental pre–post intervention study | Self-reporting questionnaires of depressive symptoms and anxiety, self-esteem, and dyadic  adjustment. | Reykjavik, Iceland within primary community health care. | 39 couples – 39 fathers  Referred by midwives for antenatal emotional distress in 2nd–3rd trimester.  Mean age: men 30 (SD 5.6).  Participation: 32% of fathers attended >2 visits; 49% attended once; 19% never attended. | Effects of an antenatal family nursing intervention for  emotionally distressed women and their partners. | Paired and independent t-tests to compare pre–post and gender differences, and repeated-measures ANOVA to assess changes over time within couples. | The Calgary Family Nursing Model is appropriate and beneficial for distressed expectant couples in primary antenatal care.  Fathers’ mental health should be routinely addressed when supporting distressed pregnant women.  The intervention was acceptable to both partners and showed positive effects for men despite lower session attendance.  Further controlled trials are needed to confirm effectiveness |
